# BP-M345 as a Basis for the Discovery of New Diarylpentanoids with Promising Antimitotic Activity

**DOI:** 10.3390/ijms25031691

**Published:** 2024-01-30

**Authors:** Joana Moreira, Patrícia M. A. Silva, Eliseba Castro, Lucília Saraiva, Madalena Pinto, Hassan Bousbaa, Honorina Cidade

**Affiliations:** 1 Laboratory of Organic and Pharmaceutical Chemistry, Department of Chemical Sciences, Faculty of Pharmacy, University of Porto, Rua de Jorge Viterbo Ferreira 228, 4050-313 Porto, Portugal; up201302558@edu.ff.up.pt (J.M.); madalena@ff.up.pt (M.P.); 2 Interdisciplinary Centre of Marine and Environmental Research (CIIMAR), University of Porto, Edifício do Terminal de Cruzeiros do Porto de Leixões, Avenida General Norton de Matos S/N, 4450-208 Matosinhos, Portugal; 3 UNIPRO—Oral Pathology and Rehabilitation Research Unit, University Institute of Health Sciences (IUCS), Cooperativa de Ensino Superior Politécnico e Universitário (CESPU), Rua Central de Gandra 1317, 4585-116 Gandra, Portugal; patricia.silva@cespu.pt (P.M.A.S.); a11230@alunos.cespu.pt (E.C.); 4 1H-TOXRUN—One Health Toxicology Research Unit, University Institute of Health Sciences (IUCS), Cooperativa de Ensino Superior Politécnico e Universitário (CESPU), Rua Central de Gandra 1317, 4585-116 Gandra, Portugal; 5 LAQV/REQUIMTE, Laboratory of Microbiology, Department of Biological Sciences, Faculty of Pharmacy, University of Porto, Rua de Jorge Viterbo Ferreira 228, 4050-313 Porto, Portugal; lucilia.saraiva@ff.up.pt

**Keywords:** BP-M345, diarylpentanoids, antitumor, mitosis

## Abstract

Recently, the diarylpentanoid **BP-M345** (**5**) has been identified as a potent in vitro growth inhibitor of cancer cells, with a GI_50_ value between 0.17 and 0.45 µM, showing low toxicity in non-tumor cells. **BP-M345** (**5**) promotes mitotic arrest by interfering with mitotic spindle assembly, leading to apoptotic cell death. Following on from our previous work, we designed and synthesized a library of **BP-M345** (**5**) analogs and evaluated the cell growth inhibitory activity of three human cancer cell lines within this library in order to perform structure–activity relationship (SAR) studies and to obtain compounds with improved antimitotic effects. Four compounds (**7**, **9**, **13**, and **16**) were active, and the growth inhibition effects of compounds **7**, **13**, and **16** were associated with a pronounced arrest in mitosis. These compounds exhibited a similar or even higher mitotic index than **BP-M345** (**5**), with compound **13** displaying the highest antimitotic activity, associated with the interference with mitotic spindle dynamics, inducing spindle collapse and, consequently, prolonged mitotic arrest, culminating in massive cancer cell death by apoptosis.

## 1. Introduction

Perturbing the mitotic spindle to interfere with cell division is one of the most successful strategies used to kill cancer cells in cancer therapy. Such perturbation is accomplished by microtubule-targeting agents (MTAs), the best characterized therapeutic drugs for the treatment of a wide range of tumor types [1,2,3,4,5,6,7]. MTAs consist of microtubule destabilizers such as vinca alkaloids (e.g., vinblastine and vincristine) and microtubule stabilizers such as taxanes (e.g., paclitaxel and docetaxel) [8,9,10,11,12,13]. Microtubule destabilizers inhibit microtubule polymerization through binding to tubulin dimers, while microtubule stabilizers inhibit microtubule dynamics through binding to tubulin polymers [14,15]. By interfering with mitotic spindle formation and dynamics, MTAs activate the spindle assembly checkpoint which, in turn, induces the prolonged arrest of dividing cells in mitosis, usually culminating in cell death [16,17,18,19,20]. Although MTAs have proven to be very effective for the treatment of cancer, their effectiveness is commonly affected by the adverse side effects associated with their long- and short-term use [21,22]. Neurological suppression, such as peripheral neuropathy, and myelosuppression resulting from the inhibition of the rapidly dividing hematopoietic cells are the main toxic side effects that occur during treatment with MTAs [3,23,24]. Also, drug resistance, namely due to efflux mechanisms associated with the overexpression of ATP-binding cassette (ABC) transporters such as P-glycoprotein (Pgp), is responsible for the poor responsiveness of tumor cells to MTAs by preventing drug accumulation within tumor cells [25,26,27]. Therefore, novel strategies and new cytotoxic agents are needed to circumvent the disadvantages associated with the MTAs currently in use in the clinic.

**Figure 1:**
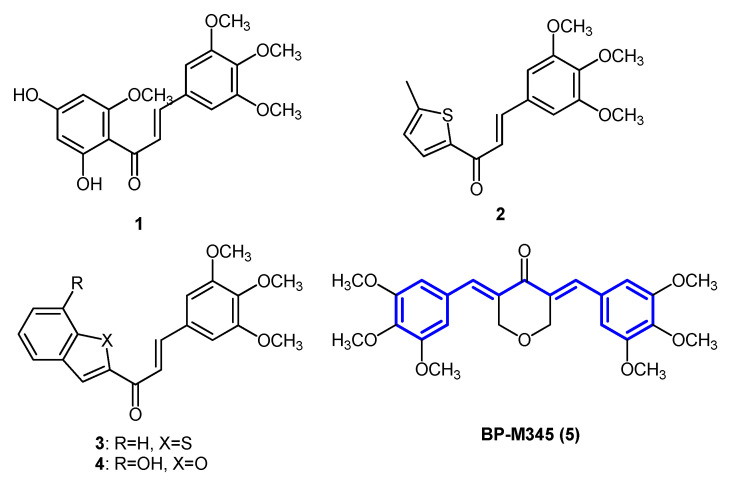
Structures of compounds (**1**–**5**) reported by our group as potential antimitotic agents. The diarylpentanoid moiety of **BP-M345** is highlighted in blue.

In recent years, several works have led to the discovery of the 3,4,5-trimethoxyphenyl fragment as a crucial moiety for the interaction with tubulin [28,29]. In fact, several chemical libraries possessing the 3,4,5-trimethoxyphenyl fragment have been developed with the aim of discovering new MTAs [28,29,30,31,32]. In this area of research, we identified the chalcone derivative **1**, as well as its structure-related synthetic analogs (**2**–**4**), all of which have a 3,4,5-trimethoxyphenyl B ring and display antiproliferative activity in several human tumor cell lines, and the growth inhibitory effect of these compounds is associated with mitotic interference [33,34] (Figure 1). Among chalcones with antitumor activity, α-substituted chalcones have proved to be quite active [35]. Particularly, the α-substitution of chalcones by alkyl groups represents a promising approach to obtain compounds with improved growth inhibitory activity in cancer cell lines through interference with microtubules [36,37]. For instance, in a recent structure–activity (SAR) relationship study performed by Sun et al. (2021), the authors concluded that α-methyl-substituted chalcones showed a higher antiproliferative activity than structure-related α-unsubstituted chalcones, with most of the antiproliferative activity being demonstrated via IC_50_ values in the sub-micromolar range [36].

Diarylpentanoids comprise a class of natural products and their synthetic analogs, which are structure-related analogs with chalcones, possessing two aromatic rings linked by a five-carbon bridge, and diarylpentanoids have gained increasing attention due to their antitumor activity [38,39,40]. Previously, our group synthesized a small library of diarylpentanoids with a tetrahydro-4*H*-pyran-4-one moiety possessing aromatic rings with different substitution patterns, including electron-withdrawing and electron-donating substituents [41]. From this library of compounds, a diarylpentanoid with two 3,4,5-trimethoxyphenyl groups, 3,5-bis((*E*-3,4,5-trimethoxybenzylidene)tetrahydro-4*H*-pyran-4-one (**BP-M345** (**5**), Figure 1), with a GI_50_ value of 0.24–0.45 µM, has been identified as a potent in vitro growth inhibitor of human cancer cells with low toxicity in normal cells, inhibiting mitosis through microtubule perturbation and causing cancer cell death, highlighting its potential as an antitumor agent [42]. Additionally, docking studies have suggested that the presence of both 3,4,5-trimethoxyphenyl groups may potentiate its inhibitory effect on tubulin [42]. It was observed that one of the 3,4,5-trimethoxyphenyl groups of **BP-M345** (**5**) occupies the colchicine binding site of α,β-tubulin, mostly buried in the β-unit, and the other 3,4,5-trimethoxyphenyl group provides extra anchoring points and strengthens the binding, occupying the α subunit of tubulin. These results suggest that the presence of both 3,4,5-trimethoxyphenyl groups could be important for a potent antimitotic effect. Nevertheless, to expand the SAR knowledge, further studies should be conducted in order to verify the effect of the linker between both groups in the antimitotic effect as well as the substitution of one of the 3-(3,4,5-trimethoxyphenyl)-prop-1-enyl groups by alkyl chains.

**Figure 2:**
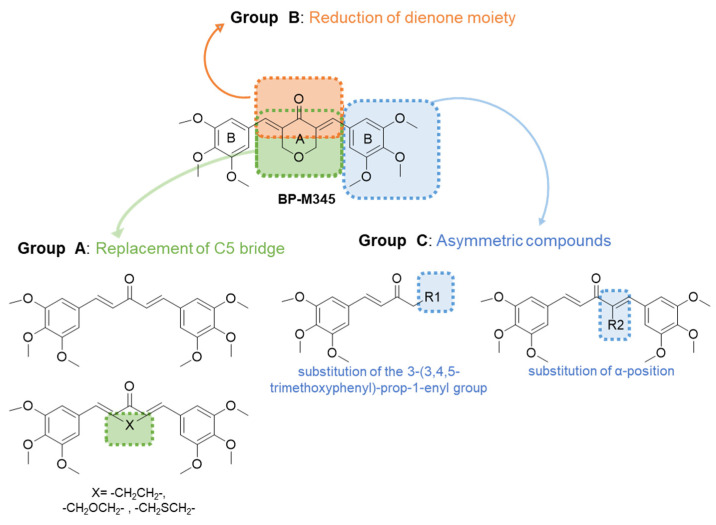
Different groups of synthetized compounds.

Following on from our previous work, in order to discover new diarylpentanoids with antimitotic activity and to perform SAR studies, we aimed to explore molecular modifications of diarylpentanoid **BP-M345** (**5**) using different strategies (Figure 2). The isosteric substitution of the tetrahydro-4*H*-pyran-4-one of **BP-M345** (**5**) by a tetrahydro-4*H*-thiopyran-4-one and cyclohexanone moieties was accomplished. Diarylpentanoids with other C5 bridges were also planned. In all cases, as a structural feature of tubulin-binding agents, 3,4,5-trimethoxy substitution in the aromatic rings was retained. Moreover, considering the homogeneity of **BP-M345** (**5**) with chalcones with only one 3,4,5-trimethoxyphenyl group with antimitotic activity, previously reported by our research group, we decided to prepare and evaluate the effects of **BP-M345** (**5**) analogs in which one of the 3-(3,4,5-trimethoxyphenyl)-prop-1-enyl groups was replaced by an alkyl chain to expand our SAR studies. Herein, the full details of the synthesis and characterization of **BP-M345** (**5**) analogs and our evaluation of **BP-M345** (**5**) and its analogs’ antiproliferative activity against three human tumor cell lines—melanoma (A375-C5), breast adenocarcinoma (MCF-7), and non-small-cell lung cancer (NCI-H460)—are presented. Furthermore, the antimitotic activity of the compounds showing potent growth inhibitory activity was characterized.

## 2. Results and Discussion

### 2.1. Synthesis

To perform SAR studies, several analogs of diarylpentanoid **BP-M345** (**5**) were planned and classified into three groups (A, B, and C), as shown in Figure 2. Group A comprised diarylpentanoids with a cyclic C5 bridge planned by isosteric substitution at position 4 of the A ring, as well as those with a cyclopentanone or with 3-oxopenta-1,4-diene moieties. With these structural modifications, we intended to evaluate the effect of chemical substituents with similar physical or chemical properties and the substitution of a cyclic bridge by an acyclic bridge in the biological activity of **BP-M345** (**5**). Group B consisted of analogs of **BP-M345** (**5**) obtained by the selective reduction of the tetrahydro-4*H*-thiopyran-4-one moiety, as we aimed to observe the impact of the enone moiety in the growth inhibitory effect. The last compound set was planned in group C. This group included **BP-M345** (**5**) analogs in which one of the 3-(3,4,5-trimethoxyphenyl)-prop-1-enyl groups was substituted by alkyl groups while retaining one 3,4,5-trimethoxyphenyl group linked to a α,β-unsaturated ketone system. With these structural modifications, we intended to evaluate the influence of different alkyl chains, including linear and branched chains, and the influence of the presence of two 3,4,5-trimethoxyphenyl groups on biological activity. Additionally, asymmetric acyclic diarylpentanoids with an alkyl chain in α-position were also planned. Despite the fact that several reports have noted the potential of α-alkyl-substituted chalcones as antitumor agents, information about the antitumor effect of α-alkyl-substituted diarylpentanoids is scarce. Thus, we set out to synthesize α-substituted diarylpentanoids to evaluate their cell growth inhibitory activity.

#### 2.1.1. Synthesis of Compounds of Group A

**Scheme 1:**
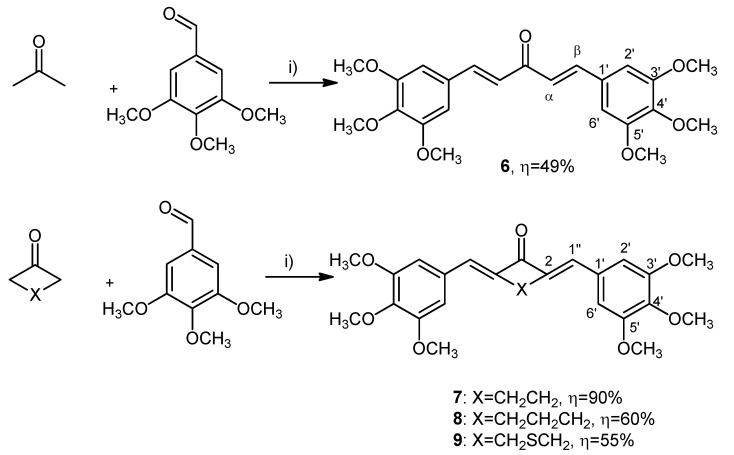
Synthesis of compounds of group A. Reaction conditions: i) 40% (*w*/*w*) NaOH solution, MeOH, reflux (the numbering used is related to the NMR assignments).

Derivatives of group A (**6**–**9**, Scheme 1) were obtained via the Claisen–Schmidt condensation of different ketones with 3,4,5-trimetoxybenzaldehyde as building blocks, with yields ranging from 49% to 90% (Scheme 1).

#### 2.1.2. Synthesis of Compounds of Group B

**Scheme 2:**
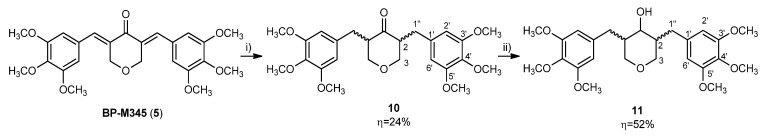
Synthesis of compounds of group B. Reaction conditions: i) Pd/C, H_2_, toluene, rt. ii) NaBH_4_, MeOH, 30 °C (the numbering used is related to the NMR assignments).

The reduction of the double bond of the α,β-unsaturated ketone system was accomplished by catalytic hydrogenation in a H_2_ atmosphere with 10% Pd/C as a catalyst and toluene as a solvent to obtain diarylpentanoid **10**. The reduction of the double bonds resulted in the disappearance of the characteristic yellow color in the precursor (Scheme 2). The confirmation of the success of the double bond reduction was accomplished by NMR. For compound **10**, instead of signals of an olephinic proton detected in the ^1^H (δ_H_ 7.77 sl, H-1″) NMR spectrum of **BP-M345** (**5**), used as a precursor, characteristic signals of benzylic protons (δ_H_ 3.18 dd (*J* = 14.3, 5.2 Hz) and 2.29 q (*J* = 7.2 Hz), H-1″a and H-1″b) and protons at the α position of the carbonyl group (δ_H_ 2.96–2.89 *m*, H-2, -6) were observed.

Additionally, the ketone moiety of **10** was further modified using sodium borohydride (NaBH_4_) to reduce the ketone to a hydroxyl group. Thus, diarylpentanoid **11** (Scheme 2) was obtained from the reduction of the carbonyl ketone moiety of compound **10** using NaBH_4_ and methanol as solvents. The reduction of the ketone group was confirmed by the appearance of a H-1 proton, obtained by the reduction of a carbonyl group at δ_H_ 3.74 (^1^H NMR), as well as by the presence of the characteristic signal C-1 instead of the signal of a carbonyl group in the precursor.

#### 2.1.3. Synthesis of Compounds of Group C

**Scheme 3:**
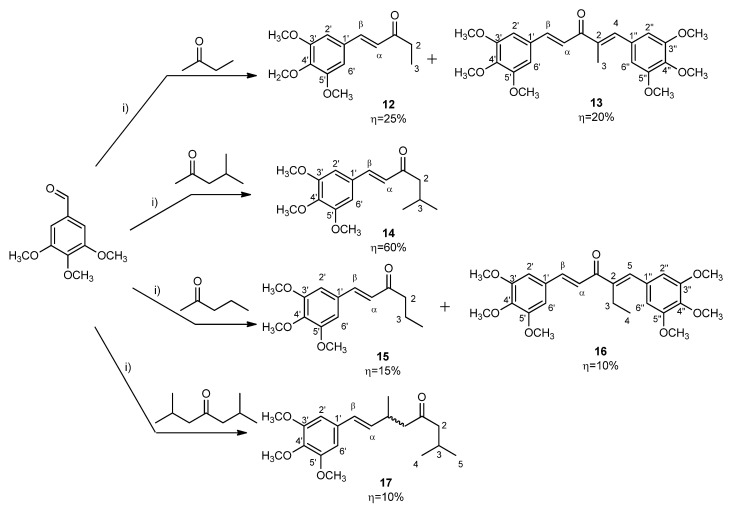
Synthesis of compounds of group C. Reaction conditions: i) 40% (*w*/*w*) NaOH solution, MeOH, r.t (the numbering used is related to the NMR assignments).

The synthesis of the compounds of group C was carried out by Claisen–Schmidt condensation, conducted at room temperature, between appropriately substituted ketones and 3,4,5-trimetoxibenzaldehyde in the presence of NaOH [41] (Scheme 3). Firstly, these reactions were performed using one equivalent of benzaldehyde to obtain planned asymmetric ketones and using two equivalents of benzaldehyde to obtain the acyclic diarylpentanoids with an α-alkyl chain as major products. However, the reactions performed with only one equivalent of benzaldehyde were incomplete, failing to give the expected asymmetric ketones as major products. Therefore, all the subsequent Claisen –Schmidt condensation reactions were carried out using two equivalents of 3,4,5-benzaldehyde. As a result, asymmetric ketones with an α,β-unsaturated system and an aliphatic chain bonded to carbonyl carbon (**12**, **14**, and **15**) were obtained with moderate yields (15–60%). When the reaction was conducted with butan-2-one or pentan-2-one, in addition to asymmetric ketones (**12** and **15**, respectively), asymmetric diarylpentanoids **13** and **16**, resulting from the aldol reaction in the α-position of ketones **12** and **15**, were also obtained as by-products; for this reason, these compounds were obtained with low yields. Interestingly, when the reaction was conducted with a symmetric ketone, 2,6-dimethylheptane-4-one, instead of the reaction occurring at the α-position, it occurred at the terminal carbon of the alkyl chain, giving rise to a ketone without α,β-unsaturation but bearing a 3,4,5-trimethoxyphenyl group (**17**).

The elucidation of the structures of compounds **5**–**9** was carried out using ^1^H and ^13^C nuclear magnetic resonance (NMR) techniques (see Figures S1–S5 in the Supplementary Materials), and the relevant data were in accordance with previously reported studies [41,43,44]. The elucidation of the structures of the new compounds **10**–**17** was carried out on the basis of NMR (see Figures S6–S13 in the Supplementary Materials) and high-resolution mass spectrometry (HRMS) (see Figures S14–S21 in the Supplementary Materials). The purity of compounds was established by HPLC (see Figures S22–S33 in the Supplementary Materials).

### 2.2. Biological Activity

#### 2.2.1. Tumor Cell Growth Inhibitory Activity Evaluation

The effect of all compounds on the in vitro growth of three human tumor cell lines—A375-C5 (melanoma), MCF-7 (breast adenocarcinoma), and NCI-H460 (non-small-cell lung cancer)—was evaluated at the maximum concentration of 75 µM according to the procedure adopted by the National Cancer Institute (NCI, Bethesda, MD, USA), which involves using the protein-binding dye sulforhodamine B (SRB) to assess cell growth, as described in the Section 3. Dose–response curves for each cell line were obtained, and the concentrations of each tested compound that caused cell growth inhibition of 50% (GI_50_) were determined (Table 1). The growth inhibitory effect of compound **17** was also tested in order to assess the potential of ketones bearing 3,4,5-trimethoxyphenyl groups without the presence of α,β-unsaturation.

As shown in Table 1, compounds **7**, **9**, **13**, and **16** were active, with GI_50_ values ranging from 0.73 to 2.09 μM in the three human tumor cell lines studied.

**Figure 3:**
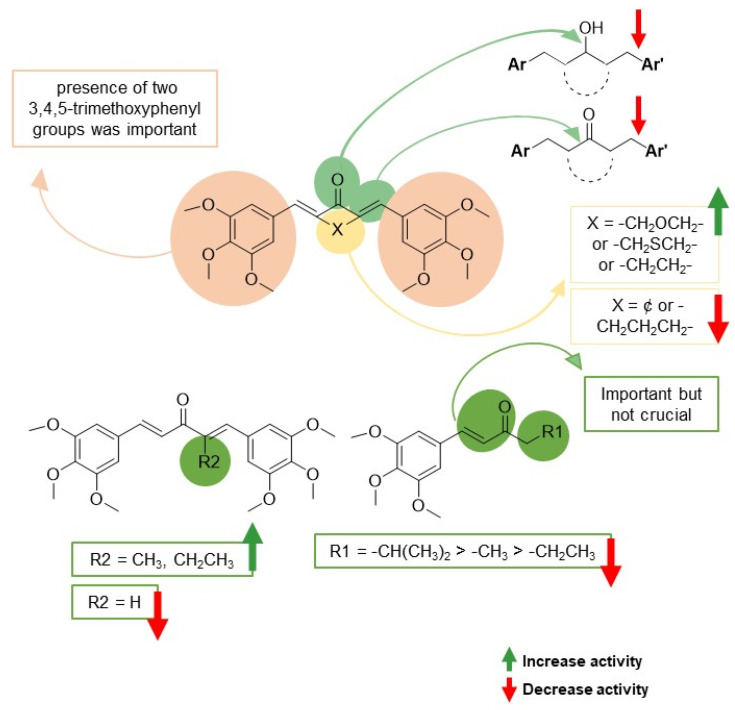
Putative SARs for synthesized **BP-M345** (**5**) analogs with antiproliferative activity.

An attempt was made to draw some considerations concerning structure–activity relationships, although the number of compounds was limited (Figure 3). For group A, when comparing the antiproliferative effects of **BP-M345** (**5**) with those of analogs **7** and **9**, although a slight decrease in activity was observed, the presence of tetrahydro-4*H*-thiopyran-4-one (**9**) or cyclopentanone (**7**) moieties was also associated with a notable growth inhibitory effect. However, the substitution of the C5 bridge by a 3-oxopenta-1,4-diene (**6**: GI_50_ > 75 µM) or a cyclohexanone moiety (**8**: GI_50_ > 75 µM) was associated with a decrease in antiproliferative activity. A similar result was observed for some compounds in group B, namely **10** and **11**, suggesting that the dienone moiety is crucial to the biological activity of **BP-M345** (**5**). Through comparing the results for ketones (**12** and **15**; GI_50_ = 18.32–67.43 µM) with structure-related diarylpentanoids with two 3,4,5-trimethoxyphenyl groups of group C (**13** and **16**; GI_50_ = 1.41–2.09 µM), we found that the presence of only one 3,4,5-trimethoxyphenyl group is not essential for antiproliferative activity, but it is associated with a high decrease in the growth inhibitory effect. These results are in accordance with the docking results previously reported by us for diarylpentanoid **BP-M345** (**5**) [42]. Interestingly, the comparison of results for ketones **12**, **14**, **15**, and **17** suggests that the presence of the α,β-unsaturated ketone moiety is important but not crucial to the antiproliferative activity. Notably, our comparison of the GI_50_ values obtained for α-substituted acyclic diarylpentanoids (**13** and **16**; GI_50_ = 1.41–2.09 µM) with the GI_50_ value of **6** (GI_50_ > 75 µM) allowed us to conclude that the α-substitution can give rise to potent compounds, as already reported for chalcones.

Since the activity of compounds **7**, **9**, **13**, and **16**, when compared to **BP-M345**, did not show enhancement, we subsequently conducted a comparison between the parental compound **BP-M345** and its derivatives to assess whether the structural changes resulted in improved antimitotic activity. Antimitotic activity is particularly significant as it targets mitosis, an indispensable step in cancer cell proliferation.

#### 2.2.2. NCI-H460 Cell Arrest in Mitosis in Response to Treatment with Compounds **7**, **13**, and **16**

**Figure 4:**
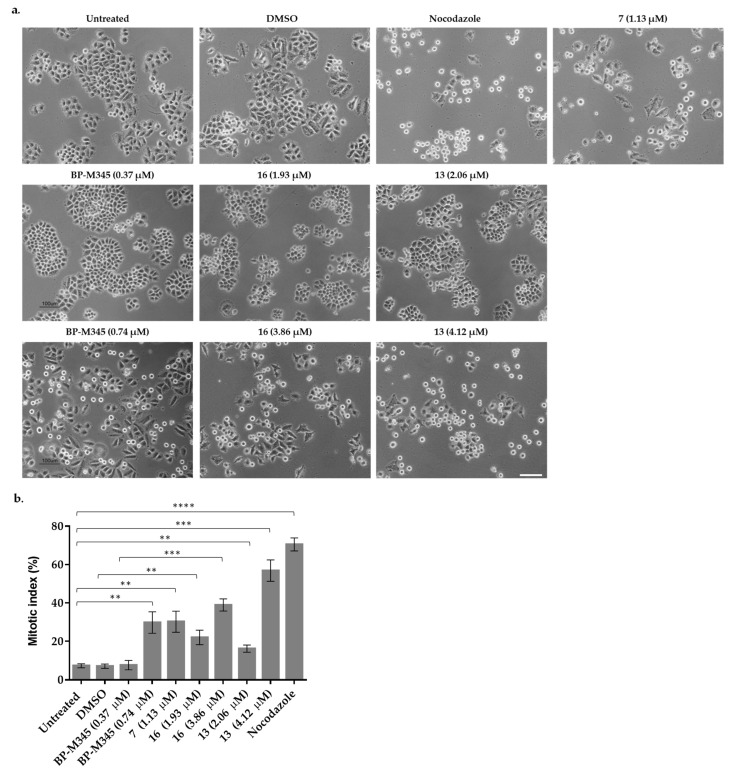
Treatment with compounds **7, 13**, and **16** leads to the mitotic arrest of cancer cells. (**a**) Representative phase-contrast microscopy images of untreated NCI-H460 cells and cells treated with **7**, **13**, and **16** compounds for 24 h at the indicated concentrations. The images highlight the presence of mitotic cells, identifiable by their rounded shape and brighter appearance compared to the others. DMSO- and nocodazole-treated cells were used as experimental controls. **BP-M345**-treated cells were also included. Bar: 100 µm. (**b**) Bar graph depicting the Mitotic index of the data presented in (**a**) demonstrating statistical relevance; ** *p* < 0.01, *** *p* < 0.001, and **** *p* < 0.0001 (determined by an unpaired *t*-test based on three independent experiments).

Diarylpentanoids **7**, **9**, **13**, and **16**, which had the lowest GI_50_ values (<2.1 µM) in the three human cancer cell lines, were tested for their potential antimitotic effect in NCI-H460 cells. For this test, the NCI-H460 cells were treated with 1 × GI_50_ and 2 × GI_50_ of each compound, except for **7**, which was used at GI_50_ because of its toxicity at higher concentrations. This approach aimed to provide a nuanced understanding of the relative antimitotic potency of each compound based on their individual activity levels. After 24 h of incubation with the compounds, the treated cells were analyzed by phase-contrast microscopy to identify the compounds that facilitated mitotic arrest. Mitotic inhibitors usually cause cell rounding, as revealed after treatment with nocodazole, used as a positive control (Figure 4a). From the four compounds tested, only compound **9** did not induce an arrest in mitosis. In contrast, **7**, **13**, and **16** were very effective in arresting the tested cancer cells in mitosis compared to untreated or DMSO-treated cells (Figure 4b). Our determination of the mitotic index (MI) revealed a significant increase in MI value in the cells treated with **7** (30.2 ± 5.5%), **13** (56.9 ± 5.6%), or **16** (38.9 ± 3.1%) compared to the untreated (7.3 ± 1.0%) or DMSO-treated (7.0 ± 1.1%) cells (Figure 4b). Of note, compound **13** exhibited the highest antimitotic activity. Interestingly, the MI after treatment with **16** and, particularly, **13** was significantly higher than the previously reported MI (29.8 ± 5.6%) induced by the parent compound **BP-M345** (**5**) when used at 2 × GI_50_ [42]. Interestingly, after treatment with concentrations corresponding to GI_50_, the MI in the **BP-M345**-treated cells was indistinguishable from that of the control cells (7.6 ± 2.4%); however, in the cells treated with compounds **13** and **16**, the MI remained significantly higher, measuring 16.2 ± 1.9% and 22.0 ± 3.8%, respectively. This clearly indicates that **BP-M345** analogs exhibit a more potent mitotic activity.

#### 2.2.3. Cancer Cells Treated with Compounds **7**, **13**, and **16** Undergo Apoptosis

**Figure 5:**
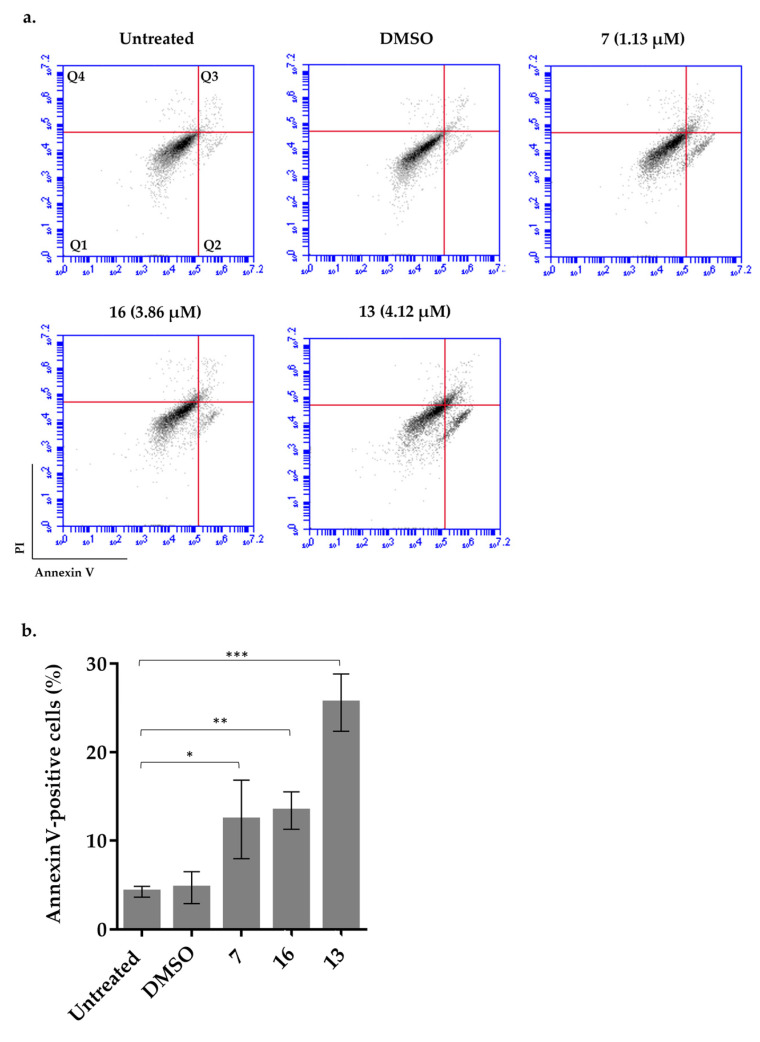
Treatment with compounds **7**, **13**, and **16** induces apoptosis in cancer cells. (**a**) Representative flow cytometry histogram of untreated cells and cells treated with compounds, stained with propidium iodide (PI) and Annexin V (FITC-A), at 24 h. Cells treated with DMSO were used as experimental controls. The quadrants (Qs) were defined as follows: Q1 = live cells (Annexin V-negative/PI-negative), Q2 = early stage of apoptosis (Annexin V-positive/PI-negative), Q3 = late stage of apoptosis (Annexin V-positive/PI-positive), and Q4 = necrosis (Annexin V-negative/PI-positive). (**b**) Quantification of AnnexinV-positive cells, based on the data shown in (**a**), demonstrating statistical relevance; * *p* < 0.05, ** *p* < 0.01, and *** *p* < 0.001 (determined by an unpaired *t*-test based on three independent experiments).

Cancer cells are expected to die after a prolonged arrest in mitosis. To verify this, in our study, the NCI-H460 cells were exposed to each compound for 24 h as above, and double-staining with annexin V-FITC and propidium iodide dyes was performed to identify treated cells undergoing apoptosis events using a flow cytometer. As shown in (Figure 5), the three compounds exhibited apoptotic induction upon being compared to the untreated or DMSO-treated control cells. Again, compound **13** was the most potent inducer of an apoptotic cancer cell death (25.6 ± 3.2% Annexin V-positive cells), being even more potent than the parent compound **BP-M345** (**5**) (16.8 ± 6.2%) [42].

**Figure 6:**
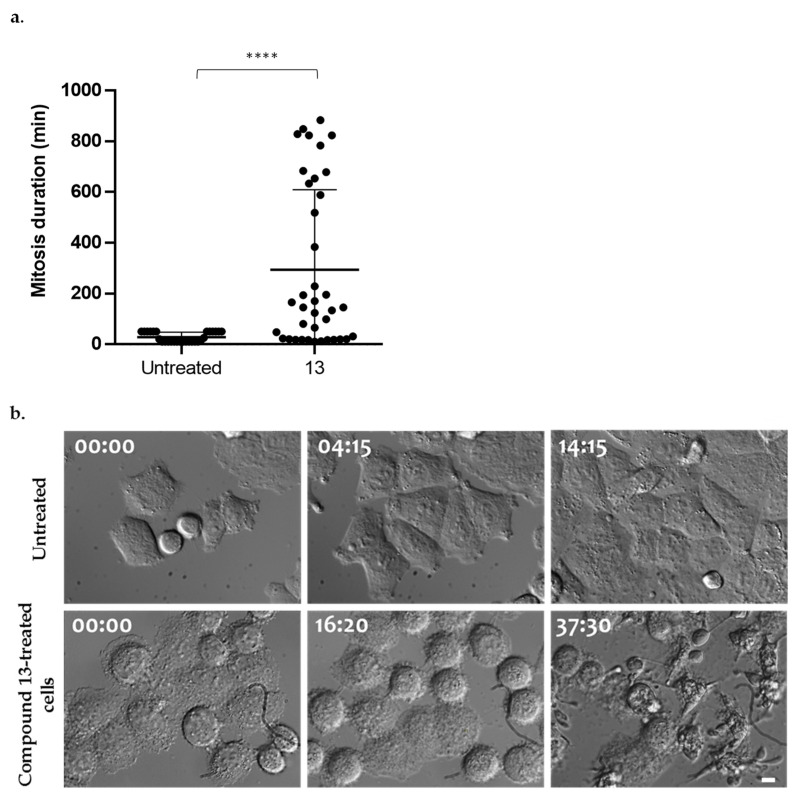
Treatment with compounds **7, 13,** and **16** induces massive cell death after a prolonged mitotic arrest. (**a**) Mitosis duration in untreated (*n* = 26) and compound **13**-treated cells (*n* = 38). **** *p* < 0.0001 (determined by an unpaired *t*-test). Each spot represents one cell. (**b**) Representative time-lapse sequences of untreated (**top**) and compound **13**-treated cells (**bottom**). Untreated cells underwent normal cycling. Compound **13**-treated cells experienced an arrest in mitosis (rounded cells, 16 h:20 min) followed by massive death in mitosis (37 h:30 min). Bar: 5 µm.

Using differential interference contrast (DIC) time-lapse microscopy to monitor the treated cells in real time in order to understand when cell death occurs, we found that cell death under compound **13** treatment occurred mainly in mitosis after a prolonged arrest of 293.51 ± 316.12 min. Almost the entire microscope field was filled by cell blebbing and debris, corresponding to cell death before the end of the imaging. In contrast, and as expected, the untreated cells completed mitosis after 34.90 ± 28.69 min, achieving multiple divisions with normal timing during the entire course of the movie (Figure 6).

Overall, the results show that the tested **BP-M345** (**5**) analogs are potent cytotoxic agents against cancer cells, exerting their antiproliferative activity by arresting cells in mitosis, thereby inducing cell death by apoptosis. Importantly, we identified **13** as a highly potent antimitotic agent that is more effective than its parent, **BP-M345** (**5**).

#### 2.2.4. Treatment with Compounds **7**, **13**, and **16** Results in Collapsed Mitotic Spindles

**Figure 7:**
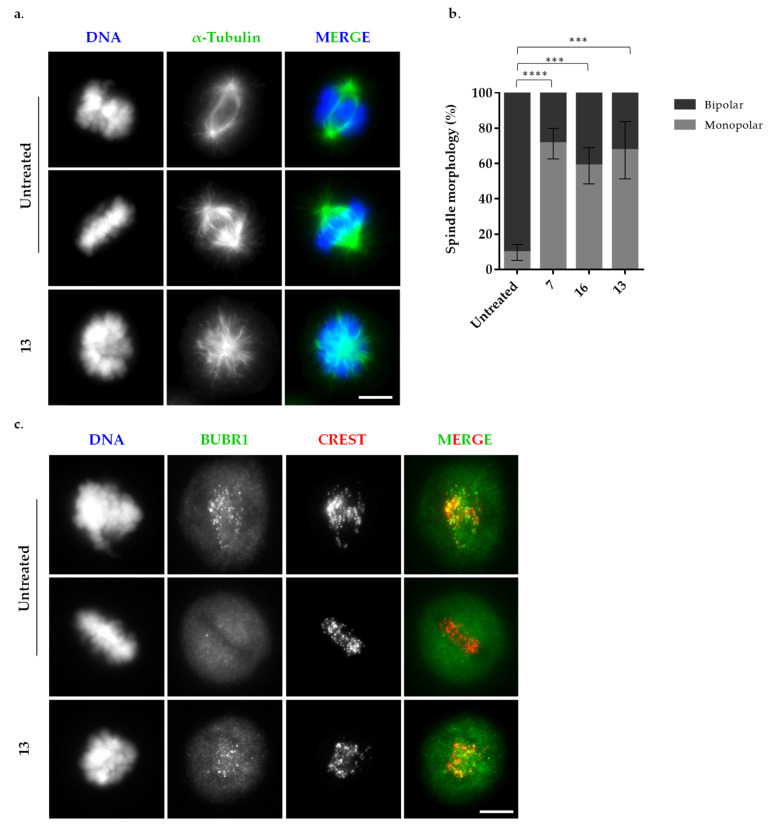
Treatment with compounds **7**, **13**, and **16** promotes mitotic spindle collapse and activates the spindle assembly checkpoint. (**a**) Representative immunofluorescence images of untreated and compound **13**-treated cells showing a bipolar spindle shape and a collapsed spindle (monopolar), respectively. The same phenotype was observed for compound **7**- and compound **16**-treated cells. The microtubules are in green, and the DNA is shown in blue. Bar: 5 µm. (**b**) Quantification of monopolar and bipolar spindles in untreated cells and compound **7**-, compound **16**-, and compound **13**-treated cells. **** *p* < 0.0001 and *** *p* < 0.001 indicate statistical significance (as determined by a two-way ANOVA with Sidak’s multiple comparisons test based on three independent experiments). (**c**) Representative immunofluorescence images of untreated and compound **13**-treated cells stained for BUBR1 (green), CREST (red), and DNA (blue). Bar: 5 µm.

To gain insights into the mechanism behind the mitotic arrest and subsequent apoptosis after treatment with compounds **7**, **13**, and **16**, and considering that **BP-M345** disturbs mitotic spindle and compromises chromosome congression, we performed an immunofluorescence assay to visualize spindle microtubules and the DNA (Figure 7a). We observed that after exposure to compounds **7**, **13**, and **16**, more than 50% of the cells exhibited abnormal spindle morphology, showing collapsed spindles resembling monopolar spindles, while in the untreated cells, this phenotype was only visible in less than 10% of the cells (Figure 7b). Additionally, we found that the cells with monopolar spindle morphologies, induced after exposure to compounds **7**, **13**, and **16**, had an activated spindle assembly checkpoint (SAC), as suggested by the presence of BUBR1 at the kinetochores in these cells (Figure 7c), a common marker of SAC activation.

## 3. Materials and Methods

### 3.1. Chemistry

All reactions were monitored by thin-layer chromatography (TLC). The purification of the compounds was carried out by flash column chromatography (CC), and preparative TLC was performed using Macherey-Nagel silica gel 60 (0.04–0.063 mm) and Macherey-Nagel silica gel 60 (GF254) plates (Düren, Germany), respectively. Melting Points were obtained in a Köfler microscope (Berlin, Germany) and are presented uncorrected. ^1^H and ^13^C NMR spectra were taken in CDCl_3_ at room temperature on Bruker Avance 300 or 400 instruments (300.13 MHz or 400.14 MHz and 75.47 MHz or 100.63 MHz MHz for ^13^C). Chemical shifts are expressed in δ (ppm) values relative to tetramethylsilane (TMS) (used as an internal reference); ^13^C NMR assignments were made via 2D (HSQC and HMBC) NMR experiments (long-range ^13^C-^1^H coupling constants were optimized to 7 Hz). Spectral treatment was performed using the MestReNova v6.0.2-5475 software. High-resolution mass spectrometry (HRMS) was carried out on an LTQ OrbitrapTM XL hybrid mass spectrometer (Thermo Fischer Scientific, Bremen, Germany) controlled by LTQ Tune Plus 2.5.5 and Xcalibur 2.1.0. at CEMUP—University of Porto, Porto, Portugal. A Combi MALDI electrospray ionization (ESI) source was used to generate ions. All reagents were obtained from Sigma-Aldrich (St. Louis, MO, USA). The following materials were synthesized and purified using the described procedures. High-Performance Liquid Chromatography (HPLC) was performed in a system with a Thermo Scientific SpectraSystem P4000 pump equipped with a degasser, a Thermo Scientific SpectraSystem AS3000 autosampler (Thermo Fisher Scientific, Waltham, MA, USA) fitted with a maximum volume 100 µL loop, and a Thermo Scientific SpectraSystem UV8000 DAD detector (Waltham, MA, USA). Data acquisition was carried out using ChromQuest 5.0 software version 3.2.1. HPLC analysis was carried out using ACE—C18 (150 × 4.6 mm I.D., particle size 5 µm), manufactured by Advanced Chromatography Technologies Ltd. (Aberdeen, Scotland, UK), and the mobile phase compositions consisted of acetonitrile and water (70:30 *v/v*) for compounds **6**–**9**, **11**, and **13–17**; acetonitrile and water (50:50 *v/v*) for compound **12**; and acetonitrile and water (70:30 *v/v;* 0.1%CH_3_CO_2_H) for compound **10** (all were HPLC-grade solvents obtained from Merck Life Science S.L.U., Darmstadt, Germany). The flow rate was 1.0 mL/min, and the UV detection wavelength was set at 254 nm or 33 nm. Analyses were carried out in isocratic mode in a 30 min run at room temperature. Peak purity index was determined by the total peak UV-Vis spectra between 210 and 650 nm with a step of 4 nm.

#### 3.1.1. Synthesis of Compounds of Group A

(1*E*,4*E*)-1,5-bis(3,4,5-trimethoxyphenyl)penta-1,4-dien-3-one (**6**), 2,5-bis((*E*)-3,4,5-trimethoxybenzylidene)cyclopentan-1-one (**7**), 2,6-bis((*E*)-3,4,5-trimethoxybenzylidene)cyclohexan-1-one (**8**), and 3,5-bis((*Z*)-3,4,5-trimethoxybenzylidene)tetrahydro-4*H*-thiopyran-4-one (**9**) were synthesized (49–90% yield) and characterized according to the procedure described in [43,44]. Experimental descriptions of compounds **6**–**9** are presented in the Supplementary Materials.

#### 3.1.2. Synthesis of Compounds of Group B

Synthesis of 3,5-bis((*E*-3,4,5-trimethoxybenzylidene)tetrahydro-4*H*-pyran-4-one (**BP-M345** (**5**))

3,5-bis((*E*-3,4,5-trimethoxybenzylidene)tetrahydro-4*H*-pyran-4-one (**BP-M345** (**5**)) was synthesized (60% yield) and characterized according to the procedure described in [41]. An experimental description of compound **BP-M345** (**5**) is presented in the Supplementary Materials.

Synthesis of 3,5-bis(3,4,5-trimethoxybenzyl)tetrahydro-4*H*-pyran-4-one (**10**)

A 25 mL round-bottom flask with a stir bar was charged with 0.2 equivalents of Pd/C (46.63 mg, 438 µmol) 10% wt, 3,5-bis((*E*)-3,4,5-trimethoxybenzyldiene)tetrahydro-4*H*-pyran-4-one (**BP-M345** (**5**), 1.00 g, 2.19 mmol), and 20 mL of toluene. The reaction mixture was stirred in a H_2_ atmosphere at room temperature for 21 h. The reaction was monitored by TLC. After completion, the reaction mixture was filtered through celite and recovered with ethyl acetate. The solvent was evaporated under reduced pressure. A mixture of products was obtained and purified by flash CC (SiO_2_; n-hexane: ethyl acetate, 4:6) and preparative TLC (SiO_2_; n-hexane:ethyl acetate, 3:7) to give a yellow gum corresponding to 3,5-bis(3,4,5-trimethoxybenzyl)tetrahydro-4*H*-pyran-4-one (**10**).

3,5-bis(3,4,5-trimethoxybenzyl)tetrahydro-4*H*-pyran-4-one (**10**). Yield: 24.3% as yellow gum; 99.9% purity; ^1^H NMR (CDCl_3_, 400.14 MHz) δ: 6.37 (s, 4H, H-2′, -6′), 4.18 (q, *J* = 5.8 Hz, 2H, H-3a), 3.83 (s, 12H, 3′-, 5′-OCH_3_), 3.81 (s, 6H, 4′-OCH_3_), 3.34 (t, *J* = 11.2 Hz, 2H, H-3b), 3.18 (dd, *J* = 14.3, 5.2 Hz, 2H, H-1″a), 2.96-2.89 (m, 2H, H-2), 2.29 (q, *J* = 7.2 Hz, 2H, H-1″b) ppm; ^13^C NMR (CDCl_3_, 100.63 MHz) δ: 208.4 (C=O), 153.3 (C-3′, -5′), 136.5 (C-4′), 135.1 (C-1′), 105.8 (C-2′, -6′), 73.9 (C-3), 61.0 (4′-OCH3), 56.2 (3′-, 5′-OCH_3_), 53.2 (C-2), 31.5 (C-1″) ppm; HRMS (ESI^+^): *m*/*z* Anal. Calc. for C_25_H_32_O_8_K (M + K^+^) 499.17288. Found 499.17157.

Synthesis of 3,5-bis(3,4,5-trimethoxybenzyl)tetrahydro-2*H*-pyran-4-ol (**11**)

A reaction was carried out in a 25 mL round-bottom flask with 3,5-bis(3,4,5- trimethoxybenzyl)tetrahydro-4*H*-pyran-4-one (**10**, 76.0 mg, 165 µmol) in 20 mL of methanol. Solid NaBH_4_ (6.87 mg, 181 µmol) was then added to the magnetically stirred solution at 30 °C. The reaction was left at room temperature for 18 h and monitored by TLC. After cooling, the reaction was poured into crushed ice and neutralized with 1 M HCl solution. The solution was extracted with ethyl acetate (3 × 50 mL). The organic phase was collected, dried over with anhydrous sodium sulfate, and concentrated under reduced pressure. The residue was purified by preparative TLC (SiO_2_; n-hexane:ethyl acetate, 4:6) to obtain 3,5-bis(3,4,5-trimethoxybenzyl)tetrahydro-2*H*-pyran-4-ol (**11**).

3,5-bis(3,4,5-trimethoxybenzyl)tetrahydro-2*H*-pyran-4-ol (**11**). Yield 52.0% as white solid, 95.4% purity; mp 190–192 °C (ethyl acetate); ^1^H NMR (CDCl_3_, 400.13 MHz) *δ*: 6.37 (s, H-2′, -6′), 3.84 (s, 1H, OH), 3.82 (s, 12H, 3′-, 5′-OCH_3_), 3.81 (s, 6H, 4′-OCH_3_), 3.74 (sl, 1H, H-1), 3.60 (q, *J* = 5.6 Hz, 2H, H-3a), 3.50 (t, *J* = 11.3 Hz, 2H, H-3b), 2.55 (dd, *J* = 13.7; 7.6 Hz, 2H, H-1″a), 2.46 (dd, *J* = 13.7; 7.8 Hz, 2H, H-1″b), 2.02-1.95 (m, 2H, H-2) ppm; ^13^C NMR (CDCl_3_, 100.63 MHz) *δ*: 153.3 (C-3′, -5′), 136.5 (C-4′), 135.2 (C-1′), 105.9 (C-2′, -6′), 67.9 (C-1), 66.5 (C-3), 61.0 (4′-OCH_3_), 56.2 (3′-, 5′-OCH_3_), 43.5 (C-2), 35.1 (C-1″) ppm; HRMS (ESI^+^): *m*/*z* Anal. Calc. for C_25_H_34_O_8_Na (M + Na ^+^) 485.21459. Found 485.21560.

#### 3.1.3. Synthesis of Compounds of Group C

##### General Procedure for the Synthesis of Compounds of Group C

An aqueous solution of 40% sodium hydroxide was added to a solution of appropriate ketone (100–200 mg, 124–248 µL, 1.39–2.00 mmol, 1 eq.) in methanol until pH 13–14. Then, 3,4,5-trimethoxybenzaldehyde (544–784 g, 2.77–3.99 mmol, 2eq.) dissolved in methanol was slowly added to the reaction mixture. The reaction was left at room temperature for 1–5 days and monitored by TLC. After, crushed ice was added to the reaction mixture and neutralized with 5 M HCl solution. The solution was then extracted with ethyl acetate (3 × 50 mL). The organic layers were collected, washed with water, dried over with anhydrous sodium sulfate, and concentrated under reduced pressure. The crude product was purified as indicated below for the referred compounds.

(*E*)-1-(3,4,5-trimethoxyphenyl)pent-1-en-3-one (**12**): Purified by flash CC (SiO_2_; dichloromethane:ethyl acetate, 9:1). Yield: 25% as white solid; 99.7% purity; mp 85–87 °C (ethyl acetate); ^1^H NMR (CDCl_3_, 400.13 MHz) *δ*: 7.47 (d, *J* = 16.2 Hz, 1H, H-β), 6.65 (d, *J* = 16.1 Hz, 1H, H-α), 6.78 (s, 2H, H-2′, -6′), 3.90 (s, 6H, 3′-, 5′-OCH_3_), 3.88 (s, 3H, 4′-OCH_3_), 2.70 (q, *J* = 7.2 Hz, 2H, H-2), 1.18 (t, *J* = 7.3 Hz, 3H, H-3) ppm; ^13^C NMR (CDCl_3_, 100.63 MHz) *δ*: 200.9 (C=O), 153.6 (C-3′, -5′), 142.8 (C-β), 139.9 (C-4′), 130.2 (C-1′), 125.6 (C-α), 105.6 (C-2′, -6′), 61.1 (4′-OCH_3_), 56.3 (3′-, 5′-OCH_3_), 34.1 (C-2), 8.4 (C-3) ppm; HRMS (ESI^+^): *m*/*z* Anal. Calc. for C_14_H_19_O_4_ (M + H^+^) 251.12779. Found 251.12833.

(1*E*,4*E*)-2-methyl-1,5-bis(3,4,5-trimethoxyphenyl)penta-1,4-dien-3-one (**13**): Purified by flash CC (SiO_2_; dichloromethane:ethyl acetate, 8:2), and the by preparative TLC (SiO_2_; n-hexane:ethyl acetate, 7:3). Yield: 20% as light-yellow solid; 97.2% purity; mp 120–123 °C (ethyl acetate); ^1^H NMR (CDCl_3_, 400.14 MHz) *δ*: 7.61 (d, *J* = 15.6 Hz, 1H, H-β), 7.49 (sl, 1H, H-4), 7.26 (d, *J* = 15.6 Hz, 1H, H-α), 6.83 (s, 2H, H-2′, -6′), 6.70 (s, 2H, H-2″, -6″), 3.91 (s, 6H, 3′-, 5′-OCH_3_), 3.90 (s, 3H, 4′-OCH_3_), 3.89 (s, 9H, 3″-, 4″-, 5″-OCH_3_), 2.22 (d, *J* = 1.4 Hz, 3H, H-3) ppm; ^13^C NMR (CDCl_3_, 100.63 MHz) *δ*: 192.8 (C=O), 153.6 (C-3′, -5′), 153.3 (C-3″, -5″), 143.8 (C-β), 140.4 (C-4′), 138.8 (C-4″), 138.7 (C-4), 138.1 (C-2), 131.6 (C-1″), 130.8 (C-1′), 121.5 (C-α), 107.3 (C-2″, -6″), 105.7 (C-2′, -6′), 61.1 (4′-, 4″-OCH_3_), 56.4 (3′-, 3″-, 5′-, 5″-OCH_3_), 14.2 (C-3) ppm; HRMS (ESI^+^): *m*/*z* Anal. Calc. for C_24_H_29_O_7_ (M + H^+^) 429.19078. Found 429.19173.

(*E*)-5-methyl-1-(3,4,5-trimethoxyphenyl)hex-1-en-3-one (**14**): Purified by CC (SiO_2_; dichloromethane:ethyl acetate, 9:1) and then by preparative TLC (SiO_2_, n-hexane:ethyl acetate, 7:3) Yield: 60% as white solid; 99.5% purity; mp 89–92 °C (ethyl acetate); ^1^H NMR (CDCl_3_, 400.14 MHz) *δ*: 7.45 (d, *J* = 16.1 Hz, 1H, H-β), 6.64 (d, *J* = 16.1 Hz, 1H, H-α), 6.77 (s, 2H, H-2′, -6′), 3.89 (s, 6H, 3′-, 5′-OCH_3_), 3.88 (s, 3H, 4′-OCH_3_), 2.53 (d, *J* = 7.0 Hz, 2H, H-2), 2.29-2.17 (m, 1H, H-3), 0.98 (d, *J* = 6.6 Hz, 6H, H-4, -5) ppm; ^13^C NMR (CDCl_3_, 100.63 MHz) *δ*: 200.3 (C=O), 153.6 (C-3′, -5′), 142.6 (C-β), 140.4 (C-4′), 130.2 (C-1′), 126.2 (C-α), 105.5 (C-2′, -6′), 61.1 (4′-OCH_3_), 56.3 (3′-, 5′-OCH_3_), 49.9 (C-2), 25.4 (C-3), 22.8 (C-4, -5) ppm; HRMS (ESI^+^): *m*/*z* Anal. Calc. for C_16_H_23_O_4_ (M + H^+^) 279.15909. Found 279.15959.

(*E*)-4-((*E*)-3,4,5-trimethoxybenzylidene)-1-(3,4,5-trimethoxyphenyl)hex-1-en-3-one (**15**): Purified by flash CC (SiO_2_; dichloromethane:ethyl acetate, 9:1) and then by TLC chromatography (SiO_2_, dichloromethane:ethyl acetate, 9:1). Yield: 15% as light-yellow solid; 97.8% purity; mp 88–90 °C (ethyl acetate); ^1^H NMR (CDCl_3_, 400.14 MHz) *δ*: 7.46 (d, *J* = 16.1 Hz, 1H, H-β), 6.64 (d, *J* = 16.1 Hz, 1H, H-α), 6.77 (s, 2H, H-2′, -6′), 3.89 (s, 6H, 3′-, 5′-OCH_3_), 3.88 (s, 3H, 4′-OCH_3_), 2.64 (t, *J* = 7.3 Hz, 2H, H-2), 1.761.66 (m, 2H, H-3), 0.98 (t, *J* = 7.4 Hz, 3H, H-4) ppm; ^13^C NMR (CDCl_3_, 100.63 MHz) *δ*: 200.5 (C=O), 153.6 (C-3′, -5′), 142.5 (C-β), 140.4 (C-4′), 130.2 (C-1′), 125.9 (C-α), 105.6 (C-2′, -6′), 61.1 (4′-OCH_3_), 56.3 (3′-, 5′-OCH_3_), 42.8 (C-2), 18.0 (C-3), 14.0 (C-4) ppm; HRMS (ESI^+^): *m*/*z* Anal. Calc. for C_25_H_31_O_7_ (M + H^+^) 443.20643. Found 443.20711.

(*E*)-4-((*E*)-3,4,5-trimethoxybenzylidene)-1-(3,4,5-trimethoxyphenyl)hex-1-en-3-one (**16**): Purified by flash CC (SiO_2_; dichloromethane:ethyl acetate, 9:1) and then by TLC chromatography (SiO_2_, dichloromethane:ethyl acetate, 9:1). Yield: 10% as light-yellow solid; 95.6% purity; mp 126–128 °C (ethyl acetate); ^1^H NMR (CDCl_3_, 400.14 MHz) *δ*: 7.56 (d, *J* = 15.6 Hz, 1H, H-β), 7.41 (sl, 1H, H-5), 7.21 (d, *J* = 15.6 Hz, 1H, H-α), 6.83 (s, 2H, H-2′, -6′), 6.67 (s, 2H, H-2″, -6″), 3.91 (s, 6H, 3′-, 5′-OCH_3_), 3.89 (s, 6H, 4′-, 4″-OCH_3_), 3.88 (s, 6H, 3″-, 5″-OCH_3_), 2.71 (q, *J* = 7.5 Hz, 2H, H-3), 1.18 (t, *J* = 7.5 Hz, 3H, H-4) ppm; ^13^C NMR (CDCl_3_, 100.63 MHz) *δ*: 192.9 (C=O), 153.6 (C-3′’, -5″), 153.3 (C-3′, -5′), 144.5 (C-2), 143.8 (C-β), 140.4 (C-4″), 139.1 (C-4′), 138.1 (C-5), 131.4 (C-1′’), 130.7 (C-1′), 122.3 (C-α), 106.8 (C-2″, -6″), 105.7 (C-2′, -6′), 61.1 (4′-, 4′’-OCH_3_), 56.4 (3′-, 5′-OCH_3_), 56.3 (3″-, 5″-OCH_3_), 20.9 (C-3), 13.7 (C-4) ppm; HRMS (ESI^+^): *m*/*z* Anal. Calc. for C_25_H_31_O_7_ (M + H^+^) 443.20643. Found 443.20711.

(*E*)-2,6-dimethyl-8-(3,4,5-trimethoxyphenyl)oct-7-en-4-one (**17**): Purified by flash CC (SiO_2_; dichloromethane:ethyl acetate, 9:1) and then by TLC chromatography (SiO_2_, n-hexane:ethyl acetate, 7:3). Yield: 10% as light-yellow solid; 99.8% purity; mp 93–96 °C (ethyl acetate); ^1^H NMR (CDCl_3_, 400.14 MHz) *δ*: 7.45 (d, *J* = 16.0 Hz, 1H, H-β), 6.64 (d, *J* = 16.1 Hz, 1H, H-α), 6.77 (s, 2H, H-2′, -6′), 3.89 (s, 6H, 3′-, 5′-OCH_3_), 3.88 (s, 3H, 4′-OCH_3_), 2.60 (dd, *J* = 15.2; 5.6 Hz, 1H, H-2″a), 2.45 (dd, *J* = 15.2; 8.2 Hz, 1H, H-2″b), 2.22-2.13 (m, 1H, H-1″), 1.69–1.62 (m, 1H, H-3), 1.17–1.11 (m, 2H, H-2), 0.92 (d, *J* = 6.6 Hz, 3H, H-3″), 0.90 (d, *J* = 4.3 Hz, 3H, H-4), 0.88 (d, *J* = 4.3 Hz, 3H, H-5) ppm; ^13^C NMR (CDCl_3_, 100.63 MHz) *δ*: 200.5 (C=O), 153.6 (C-3′, -5′), 142.5 (C-β), 140.4 (C-4′), 130.2 (C-1′), 126.3 (C-α), 105.6 (C-2′, -6′), 61.1 (4′-OCH_3_), 56.3 (3′-, 5′-OCH_3_), 48.7 (C-2′’), 46.8 (C-2), 27.8 (C-1″), 25.4 (C-3), 23.4 (C-4), 22.3 (C-5), 20.2 (C-3″) ppm; HRMS (ESI^+^): *m*/*z* Anal. Calc. for C_19_H_29_O_4_ (M + H^+^) 321.20604. Found 321.20654.

### 3.2. Biological Activity

#### 3.2.1. Cell Culture

The three human cancer cell lines used in this study were obtained from the European Collection of Cell Culture, UK, and included A375-C5 (derived from melanoma), MCF-7 (derived from breast adenocarcinoma), and NCI-H460 (derived from non-small-cell lung cancer). The choice of these three cell lines was based on their established utility as experimental models in cancer research, particularly within our research group, thereby bolstering the reliability and comparability of our results. All cell lines were grown in RPMI-1640 medium (Roswell Park Memorial Institute, Biochrom, Cambridge, UK), supplemented with 5% inactivated FBS (fetal bovine serum, Biochrom), and maintained in a humidified atmosphere (Hera Cell, Heraeus, Hanau, Germany) at 37 °C with a 5% CO_2_.

#### 3.2.2. Sulforodamine B (SRB) Assay

A total of 5.0 × 10^4^ cells were seeded into two 96-well plates containing complete medium and incubated for 24 h at 37 °C and 5% of CO_2_. One of the seeded 96-well plates was used to quantify the cell amount at time zero (plate T0). The cells were fixed with 50% (*w/v*) trichloroacetic acid (Merck Millipore, Darmstadt, Germany) for 1 h at 4 °C, washed with distilled water, and allowed to dry. The cells in the other 96-well plate were treated with two-fold dilutions of each compound, ranging from 0 to 1.17 µM, or with Doxorubicin (a positive control), ranging from 0 to 0.07 µM. After 48 h, the cells were processed as for plate T0. Then, Sulforhodamine B (Sigma-Aldrich Co., Ltd., Gillingham, UK) dye was added and incubated for 30 min at room temperature, followed by five washes with 1% (*v/v*) acetic acid (Merck Millipore, Darmstadt, Germany). The SRB protein complexes were subsequently solubilized for 30 min using 10 mM Tris buffer (Sigma-Aldrich Co., Ltd., Gillingham, UK). Using a microplate reader (Biotek Synergy 2, BioTek Instruments, Inc., Winooski, VT, USA), absorbance was measured at 515 nm. Dose–response curves for each cell line/compound were obtained, as well as for Doxorubicin, and the concentrations causing a 50% inhibition of cell growth (GI_50_) were determined. Doxorubicin was used as a positive control for the cytotoxic/viability assay.

#### 3.2.3. Mitotic Index Determination

For our determination of the mitotic index, 9.0 × 10^5^ NCI-H460 cells were initially seeded into 6-well plates containing complete culture medium and left to incubate for 24 h. Subsequently, the cells underwent a 16 h treatment period with concentrations ranging from 1 to 2 times the GI50 of each test compound, or they were treated with 1 μM of Nocodazole (Sigma-Aldrich Co., Ltd., Gillingham, UK), which served as the positive control. Additionally, dimethyl sulfoxide (DMSO) up to a concentration of 0.25% was included as a compound solvent control. A minimum of 3000 cells were observed and counted randomly under a phase-contrast microscope to determine the mitotic index (MI). The MI was computed using the following formula: MI (%) = (number of mitotic cells/total number of cells) × 100 [45]. Representative phase-contrast microscopy images were captured using a Nikon TE 2000-U microscope (Nikon, Amsterdam, The Netherlands) equipped with a DXM1200F digital camera and Nikon ACT-1 software, version 2.62 (Melville, NY, USA) using 10× magnification.

#### 3.2.4. Apoptosis Analysis

NCI-H460 cells were seeded following the same procedure used for determining the mitotic index. After 24 h of treatment with the test compounds, both the cells suspended in the medium, and those attached to the plate surface were gathered and subjected to processing using the “Annexin V-FITC Apoptosis Detection Kit” (eBioscience, Vienna, Austria) [46,47] according to the manufacturer’s instructions. Apoptosis rates were determined by flow cytometry (BD Accuri™ C6 Plus Flow cytometer, BD Biosciences, Qume Drive, San Jose, CA, USA), and data were analyzed using BD Accuri TM C6 Plus software version 1.0.27.1 At least 20,000 events per sample were collected. The data were obtained from three independent experiments.

#### 3.2.5. Live-Cell Imaging

For our live-cell imaging assays, 1.9 × 10^5^ NCI-H460 cells were grown onto LabTek II chambered cover glass (Nunc, Penfield, NY, USA) containing complete culture medium for 24 h at 37 °C with 5% CO_2_. Later, specifically 24 h later, the cells were treated with compound **13**, and images were captured at 5 min intervals up to 48 h under differential interference contrast (DIC) microscopy with 63× magnification on an Axio Observer Z.1 SD inverted microscope equipped with an incubation chamber at 37 °C and with 5% CO_2_. Movies were generated from the time-lapse images using ImageJ software, version 1.51 [45,48].

#### 3.2.6. Immunofluorescence

NCI-H460 cells growing on poly-L-lysine-coated coverslips for 24 h were incubated with compounds **7**, **13**, and **16**. After 24 h, to visualize spindle microtubules, the cells were fixed in cold methanol for 10 min at −20 °C. After being washed twice with PBS, the cells were blocked in 10% FBS in PBST (0.05% Tween in PBS) for 30 min, and then the cells were incubated for 1 h at room temperature with 1:2500 mouse anti-a-tubulin antibody (clone B-5-1-2, Sigma-Aldrich) in PBST with 5% FBS. Subsequently, the cells were fixed in 2% (*w/v*) paraformaldehyde in PBS for 12 min. After washing in PBS, the cells were permeabilized in 0.5% (*v/v*) Triton-X-100 in PBS for 7 min, blocked with 10% FBS in PBST for 30 min, and incubated for 1 h at room temperature with 1:400 mouse anti-BUBR1 antibody (Chemicon International, Temecula, CA, USA) and 1:4000 human anti-CREST (gift from E. Bronze-da-Rocha, University of Porto, Portugal). The primary antibodies were diluted in PBST containing 5% FBS. After rinsing in PBST, the cells were incubated for 1 h with the appropriate Alexa Fluor 488 and 568 conjugated secondary antibodies (Molecular Probes, Eugene, OR, USA) before being diluted 1:1500 in PBST with 5% FBS. Coverslips were mounted in a 2 µg/mL 40,6-diamidino-2-phenylindole (DAPI)-containing Vectashield and observed under a fluorescence microscope (Axio Observer Z.1 SD inverted microscope, Carl Zeiss, Oberkochen, Germany).

#### 3.2.7. Statistical Analyses

Statistical analyses were performed using either an unpaired Student’s *t*-test or a two-way ANOVA followed by Tukey’s multiple comparisons test in GraphPad Prism version 6 (GraphPad software Inc., San Diego, CA, USA). Results are expressed as the mean ± standard deviation (SD) derived from three independent experiments. Statistical significance was determined using * *p* < 0.05, ** *p* < 0.01, *** *p* < 0.001, and **** *p* < 0.0001 as indicators of the levels of significance.

## 4. Conclusions

In order to carry out the structural optimization of **BP-M345** (**5**) and SAR studies, a small library of compounds was planned, synthesized, and evaluated for their antiproliferative activity in three human cancer cell lines. Four compounds (**7**, **9**, **13**, and **16**) displayed low GI_50_ values (0.73–2.09 μM), and the growth inhibitory activity of diarylpentanoids **7**, **13**, and **16** was associated with a pronounced arrest in mitosis. These compounds, particularly compound **13**, which exhibited the highest antimitotic activity, were very effective in arresting the tested cancer cells in mitosis. Interestingly, this compound (**13**) was revealed to be a highly potent antimitotic agent that is more effective than its parent **BP-M345** (**5**). Regarding the molecular mechanisms of action of compounds **7**, **13**, and **16**, as antimitotic agents, we showed that **BP-M345** analogs interfere with mitotic spindle dynamics, inducing spindle collapse and, consequently, a prolonged mitotic arrest dependent on the spindle assembly checkpoint, which culminates in massive cancer cell death by apoptosis. Importantly, this phenotype was shown to be more aggressive than its parent, **BP-M345** (**5**), indicating a more prominent cytotoxic effect. Interestingly, all compounds with the highest antimitotic activity have two 3,4,5-trimethoxyphenyl groups, as is the case in **BP-M345**, which suggests that the presence of both groups could be important for this activity. Nevertheless, other molecular modifications will be made in the future to complete our SAR studies and to evaluate the effects of other structural modifications on antimitotic activity.

## Figures and Tables

**Table 1:** Growth inhibitory effect of compounds **2**–**13** on human tumor cell lines.

		GI_50_ (μM) ^1^	
	A375-C5	MCF-7	NCI-H460
**6**	>75	>75	>75
**7**	0.73 ± 0.07	1.33 ± 0.54	1.13 ± 0.23
**8**	>75	>75	>75
**9**	0.98 ± 0.04	0.78 ± 0.34	1.04 ± 0.07
**10**	>75	>75	>75
**11**	>75	>75	>75
**12**	48.14 ± 9.08	67.43 ± 6.80	56.79 ± 1.08
**13**	1.41 ± 0.51	2.02 ± 0.18	2.06 ± 0.75
**14**	14.57 ± 0.21	33.63 ± 3.63	29.53 ± 2.09
**15**	18.32 ± 3.99	42.29 ± 10.27	33.14 ± 0.88
**16**	1.66 ± 0.15	2.09 ± 0.42	1.93 ± 0.77
**17**	15.81 ± 0.20	15.64 ± 5.64	23.29 ± 5.74
**Doxorubicin**	0.027 ± 0.004	0.031 ± 0.004	0.023 ± 0.004
**BP-M345** (**5**) *	0.24 ± 0.01	0.45 ± 0.06	0.37 ± 0.00

^1^ GI_50_ represents the concentration that causes 50% cell growth inhibition at 48 h. Doxorubicin was used as a positive control. The results are expressed as mean ± standard deviation from three independent experiments. * Data of parent compound previously reported by Novais et al. [42].

## Data Availability

Data is contained within the article and Supplementary Materials.

## Supplementary Materials

The following supporting information can be downloaded at: https://www.mdpi.com/article/10.3390/ijms25031691/s1.
