# Supplementary material for: BP-M345 as a Basis for the Discovery of New Diarylpentanoids with Promising Antimitotic Activity
Source: Int J Mol Sci. 2024 Jan 30;25(3):1691. doi: 10.3390/ijms25031691 (PMC10855865; doi:10.3390/ijms25031691)
Supplement: Supplementary file 1 [file ijms-25-01691-s001.zip › ijms-2823245-supplementary.pdf]

Supporting Information

BP-M345 as a Basis for the Discovery of New Diarylpentanoids with Promising Antimitotic Activity

Joana Moreira [1,2,†], Patrícia M. A. Silva [3,4,†], Eliseba Castro [3], Lucília Saraiva [5], Madalena Pinto [1,2], Hassan Bousbaa [3,*] and Honorina Cidade [1,2,*]

[1] Laboratory of Organic and Pharmaceutical Chemistry, Department of Chemical Sciences, Faculty of Pharmacy, University of Porto, Rua de Jorge Viterbo Ferreira 228, 4050-313 Porto, Portugal

[2] Interdisciplinary Centre of Marine and Environmental Research (CIIMAR), University of Porto, Edifício do Terminal de Cruzeiros do Porto de Leixões, Avenida General Norton de Matos, S/N, 4450-208 Matosinhos, Portugal

[3] UNIPRO—Oral Pathology and Rehabilitation Research Unit, University Institute of Health Sciences (IUCS), Cooperativa de Ensino Superior Politécnico e Universitário (CESPU), Rua Central de Gandra 1317, 4585-116 Gandra, Portugal

[4] 1H-TOXRUN—One Health Toxicology Research Unit, University Institute of Health Sciences (IUCS), Cooperativa de Ensino Superior Politécnico e Universitário (CESPU), Rua Central de Gandra 1317, 4585-116 Gandra, Portugal

[5] LAQV/REQUIMTE, Laboratory of Microbiology, Department of Biological Sciences, Faculty of Pharmacy, University of Porto, Rua Jorge Viterbo Ferreira 228, 4050-313 Porto, Portugal

* Correspondence: hassan.bousbaa@iucs.cespu.pt (H.B.); hcidade@ff.up.pt (H.C.); Tel.: +351-224157186 (H.B.); +351-220428688 (H.C.)

† These authors contributed equally to this work.

Table of Contents

List of Figures 3

Experimental of compounds of group A 5

NMR spectra 7

HRMS spectra 20

HPLC chromatograms 28

List of Figures

Figure S1. ^{1}H and ^{13}C NMR spectra of compound **BP-M345** (**5**)... 7
Figure S2. ^{1}H and ^{13}C NMR spectra of compound **6**.. 8
Figure S3. ^{1}H and ^{13}C NMR spectra of compound **7**.. 9
Figure S4. ^{1}H and ^{13}C NMR spectra of compound **8**..10
Figure S5. ^{1}H and ^{13}C NMR spectra of compound **9**..11
Figure S6. ^{1}H and ^{13}C NMR spectra of compound **10**..12
Figure S7. ^{1}H and ^{13}C NMR spectra of compound **11**..13
Figure S8. ^{1}H and ^{13}C NMR spectra of compound **12**..14
Figure S9. ^{1}H and ^{13}C NMR spectra of compound **13**..15
Figure S10. ^{1}H and ^{13}C NMR spectra of compound **14**..16
Figure S11. ^{1}H and ^{13}C NMR spectra of compound **15**..17
Figure S12. ^{1}H and ^{13}C NMR spectra of compound **16**..18
Figure S13. ^{1}H and ^{13}C NMR spectra of compound **17**..19
Figure S14. HRMS spectrum of compound **10**. 20
Figure S15. HRMS spectrum of compound **11**. 21
Figure S16. HRMS spectrum of compound **12**. 22
Figure S17. HRMS spectrum of compound **13**. 23
Figure S18. HRMS spectrum of compound **14**. 24
Figure S19. HRMS spectrum of compound **15**. 25
Figure S20. HRMS spectrum of compound **16**. 26
Figure S21. HRMS spectrum of compound **17**. 27
Figure S22. HPLC chromatograms of compound **6**. 28
Figure S23. HPLC chromatograms of compound **7**. 29
Figure S24. HPLC chromatograms of compound **8**. 30
Figure S25. HPLC chromatograms of compound **9**. 31
Figure S26. HPLC chromatograms of compound **10**. 32
Figure S27. HPLC chromatograms of compound **11**. 33
Figure S28. HPLC chromatograms of compound **12**. 34
Figure S29. HPLC chromatograms of compound **13**. 35

Figure S30. HPLC chromatograms of compound **14**. 36
Figure S31. HPLC chromatograms of compound **15**. 37
Figure S32. HPLC chromatograms of compound **16**. 38
Figure S33. HPLC chromatograms of compound **17**. 39

Experimental of compounds of group A

An aqueous solution of 40% sodium hydroxide was added to a solution of appropriate ketone (200 mg, 1.72-4.44 mmol, 1 eq.) in methanol until pH 13-14. Then, a solution of 3,4,5-trimethoxybenzaldehyde (1.01-1.10 g, 5.17-10.7 mmol, 3eq.) in methanol was slowly added to the reaction mixture. The reaction was left at room temperature for 2-7 days and was monitored by TLC. After, crushed ice was added to the reaction mixture and neutralized with 5M HCl solution. A precipitate was formed upon cooling to room temperature. This was filtrated, washed with water, and purified as indicated below for the referred compounds.

(1*E*,4*E*)-1,5-bis(3,4,5-trimethoxyphenyl)penta-1,4-dien-3-one (**6**): Purified by crystallization from methanol. Yield: 49% as yellow solid; 99.5% purity; mp 127-129 °C (methanol); ^{1}H NMR ($CDCl_3$, 300.13 MHz) *δ*: 7.66 (d, *J* = 15.8 Hz, 2H, H-β), 6.98 (d, *J* = 15.8 Hz, 2H, H-α), 6.85 (s, 4H, H-2', -6'), 3.93 (s, 6H, 3'-, 5'-OCH_3), 3.90 (s, 3H, 3'-, 5'-OCH_3) ppm; ^{13}C NMR ($CDCl_3$, 75.47 MHz) *δ*: 188.6 (C=O), 153.6 (C-3', -5'), 143.5 (C-β), 140.5 (C-4'), 130.4 (C-1'), 124.9 (C-α), 105.7 (C-2', -6'), 61.2 (4'-OCH_3), 56.3 (3'-, 5'-OCH_3) ppm.

2,5-bis((*E*)-3,4,5-trimethoxybenzylidene)cyclopentan-1-one (**7**): Purified by crystallization from methanol. Yield: 90% as yellow solid; 99.5% purity; mp 203-205 ºC (methanol); ^{1}H NMR ($CDCl_3$, 300.13 MHz) *δ*: 7.52 (sl, 2H, H-1''), 6.84 (s, 4H, H-2', -6'), 3.91 (s, 6H, 3'-, 5'-OCH_3), 3.90 (s, 3H, 4'-OCH_3) 3.14 (sl, 4H, H-3) ppm; ^{13}C NMR ($CDCl_3$, 75.47 MHz) *δ*: 196.0 (C=O), 153.4 (C-2', -6'), 139.7 (C-4'), 136.2 (C-2), 134.2 (C-1''), 131.5 (C-1'), 61.1 (4'-OCH_3), 56.3 (3'-, 5'-OCH_3), 26.6 (C-3) ppm.

2,6-bis((*E*)-3,4,5-trimethoxybenzylidene)cyclohexan-1-one (**8**): Purified by crystallization from methanol. Yield: 60% as yellow solid; 98.9% purity; mp 196-198 ˚C (methanol); ^{1}H NMR ($CDCl_3$, 300.13 MHz) *δ*: 7.72 (sl, 2H, H-1''), 6.71 (s, 4H, H-2', -6'), 3.89 (s, 6H, 3'-, 5'-OCH_3), 3.88 (s, 3H, 4'-OCH_3), 2.95 (td, *J* = 6.2; 2.0 Hz, 4H, H-3), 1.34 (quintet, *J* = 6.0 Hz, 2H, H-4) ppm; ^{13}C NMR ($CDCl_3$, 75.47 MHz) *δ*: 190.1 (C=O), 153.1 (C-3', -5'), 138.9 (C-4'), 137.3 (C-1''), 135.5 (C-2), 131.6 (C-1'), 108.0 (C-2', -6'), 61.1 (4'-OCH_3), 56.3 (3'-, 5'-OCH_3), 28.6 (C-3), 23.1 (C-4) ppm.

3,5-bis((*Z*)-3,4,5-trimethoxybenzylidene)tetrahydro-4*H*-thiopyran-4-one (**9**): Purified by crystallization from methanol. Yield: 55% as yellow solid; 98.4% purity; mp 219-222 ˚C (methanol); ^{1}H NMR ($CDCl_3$, 300.13 MHz) *δ*: 7.71 (sl, 2H, H-1''), 6.63 (s, 4H, H-2', -6'), 3.89 (s, 3H, 4'-OCH_3), 3.88 (s, 6H, 3'-, 5'-OCH_3), 3.95 (sl, 4H, H-3) ppm; ^{13}C NMR ($CDCl_3$, 75.47 MHz) *δ*: 188.9 (C=O),

153.3 (C-3’, -5’), 139.1 (C-4’), 137.3 (C-1’’), 133.4 (C-2), 130.7 (C-1’), 107.6 (C-2’, -6’), 61.1 (4’-OCH_3), 56.4 (3’-, 5’-OCH_3), 30.2 (C-3,) ppm.

NMR spectra

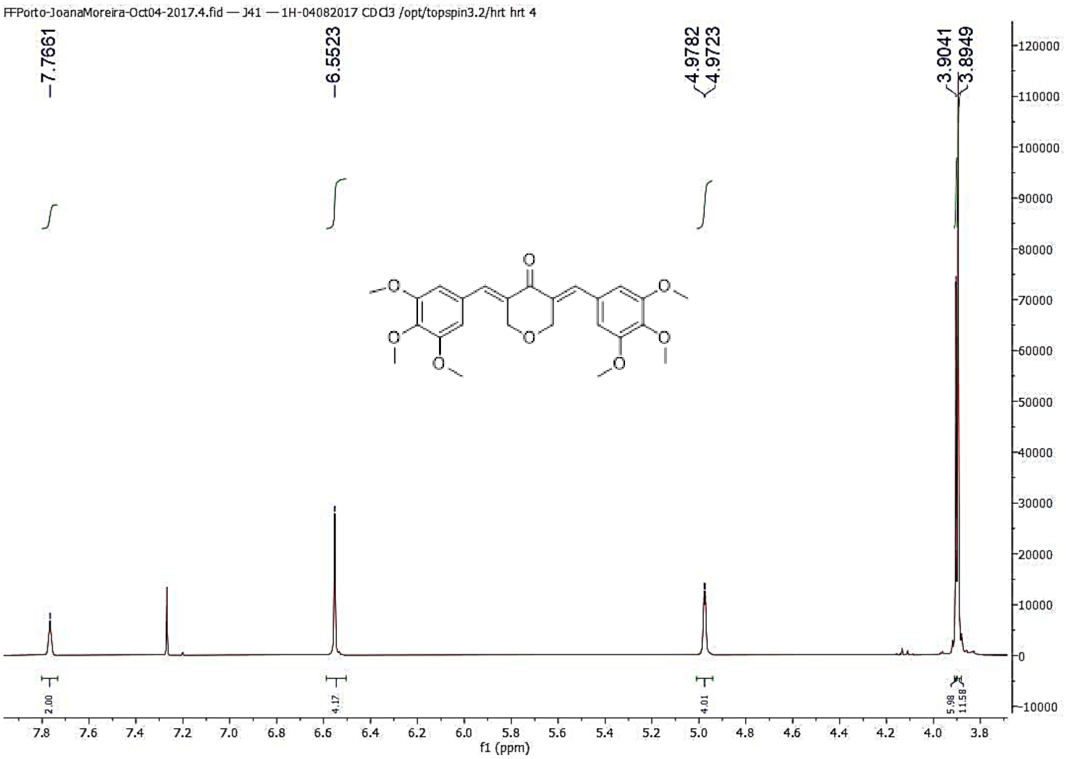

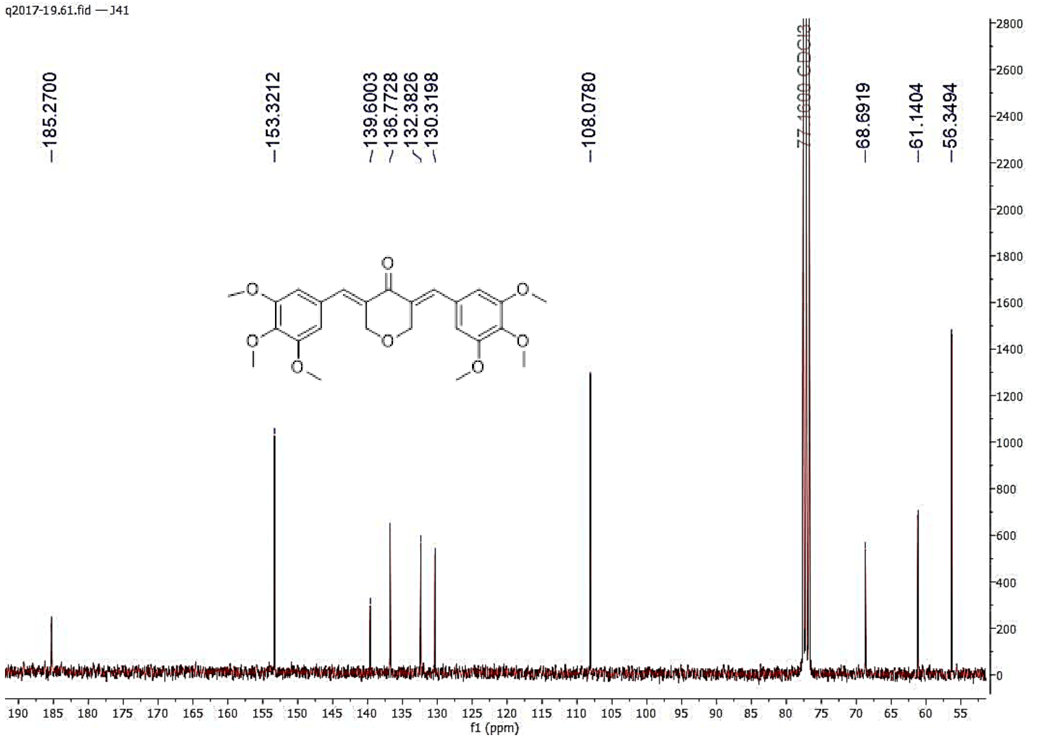

Figure S1. ^{1}H and ^{13}C NMR spectra of compound **BP-M345** (**5**).

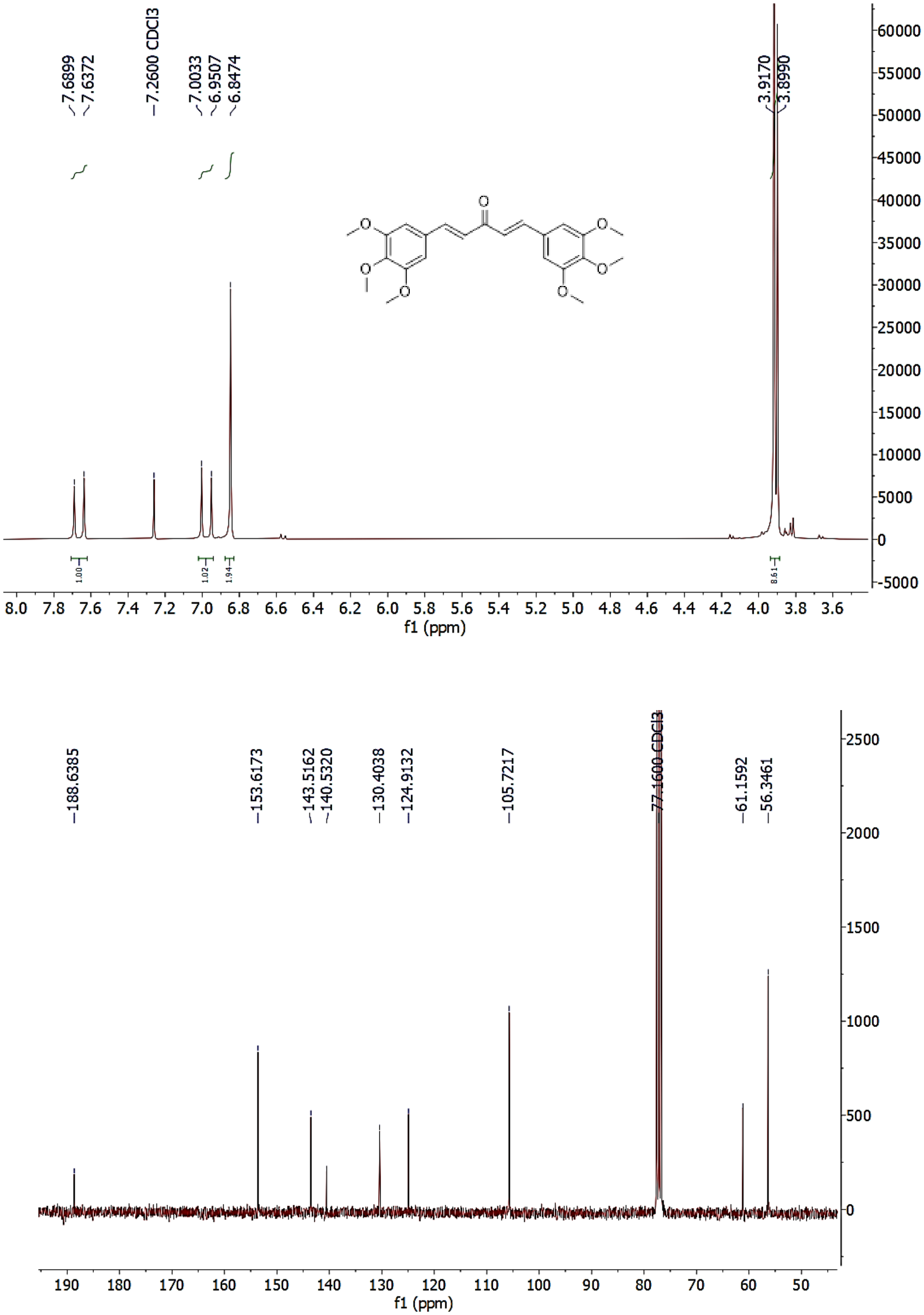

Figure S2. ^{1}H and ^{13}C NMR spectra of compound **6**.

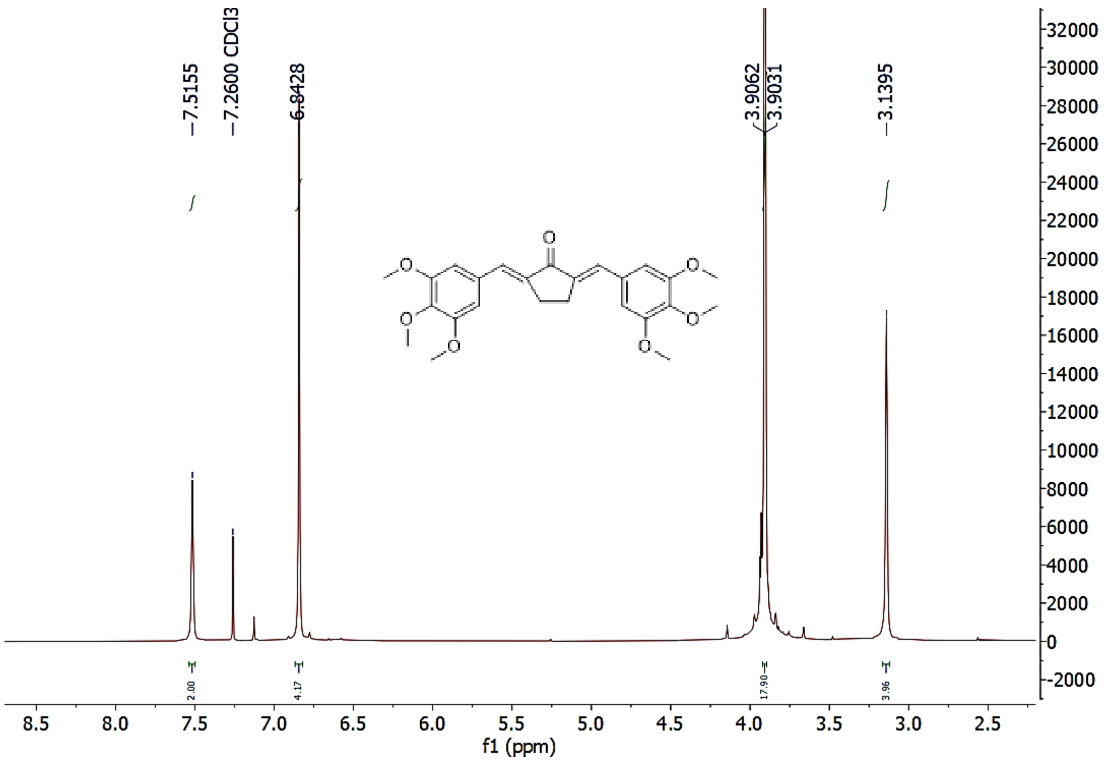

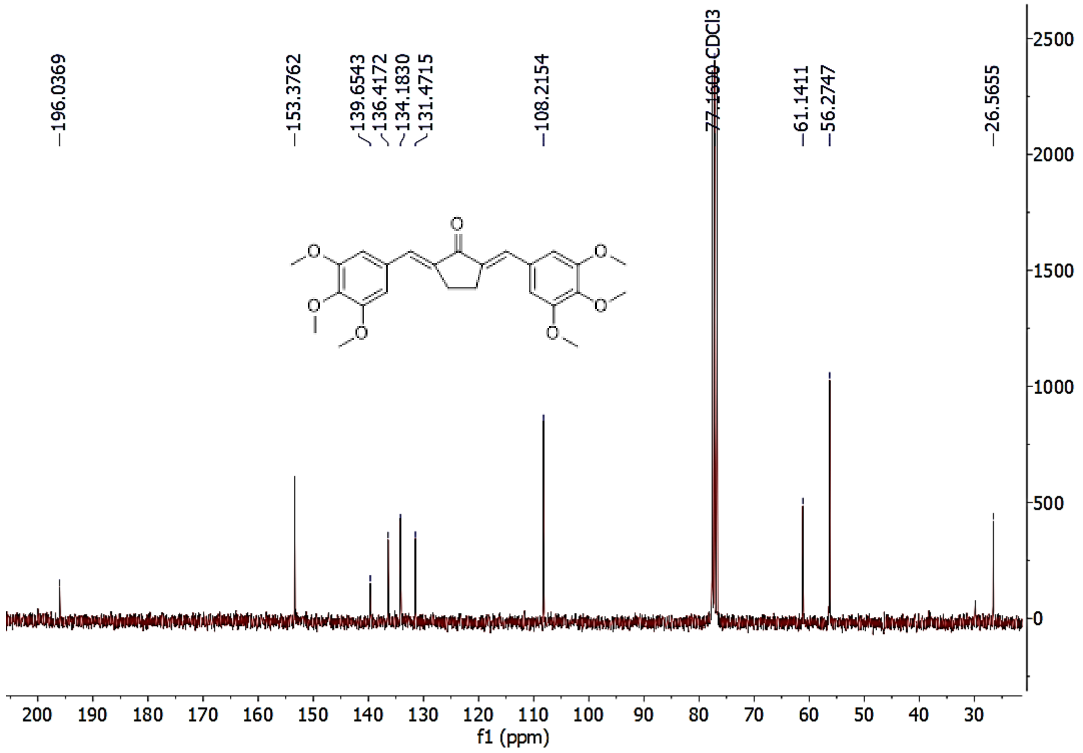

Figure S3. ^{1}H and ^{13}C NMR spectra of compound **7**.

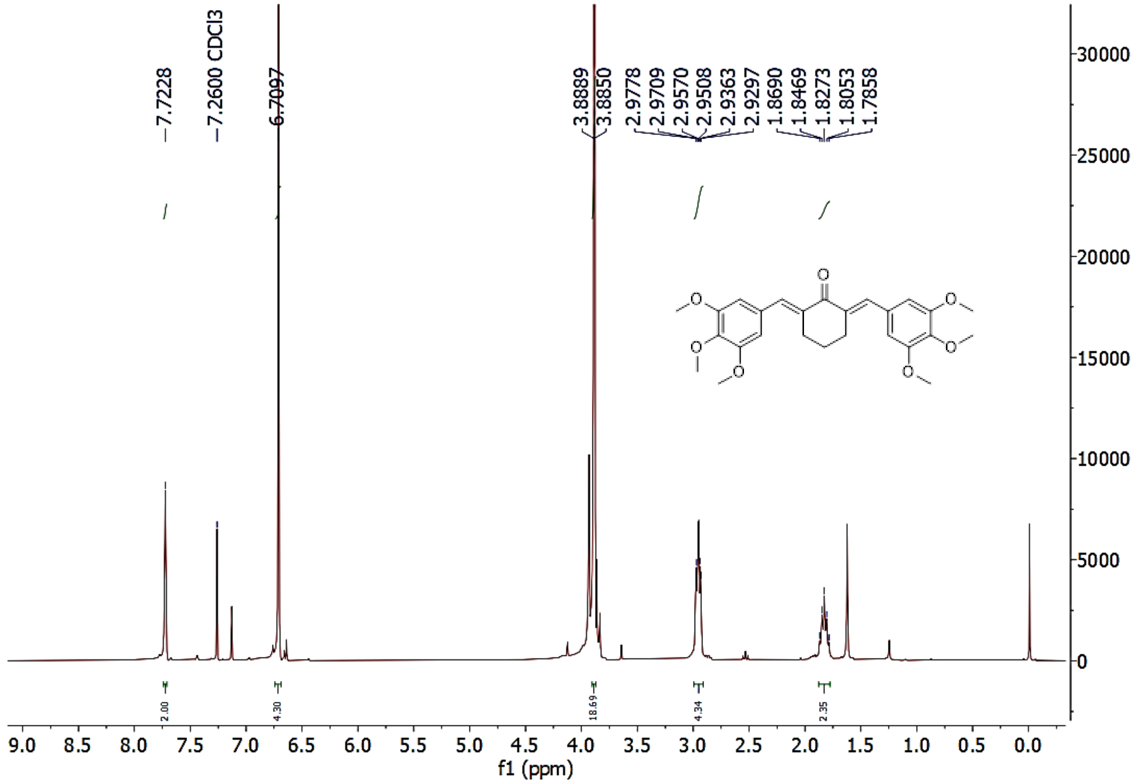

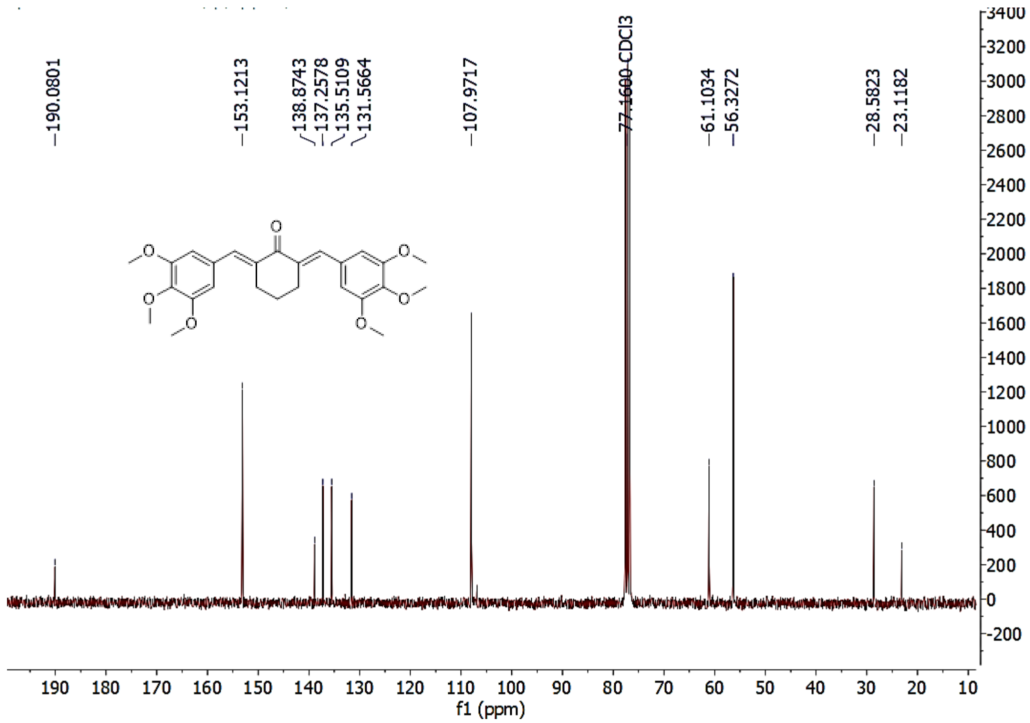

Figure S4. ^{1}H and ^{13}C NMR spectra of compound **8**.

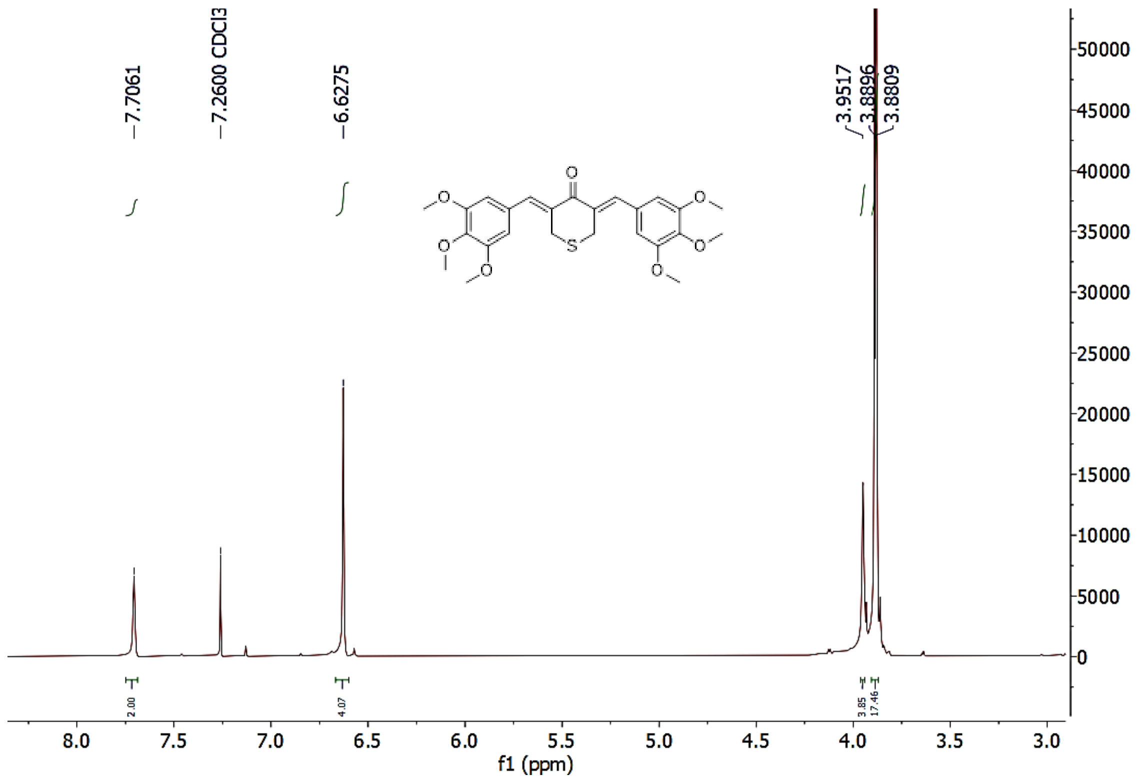

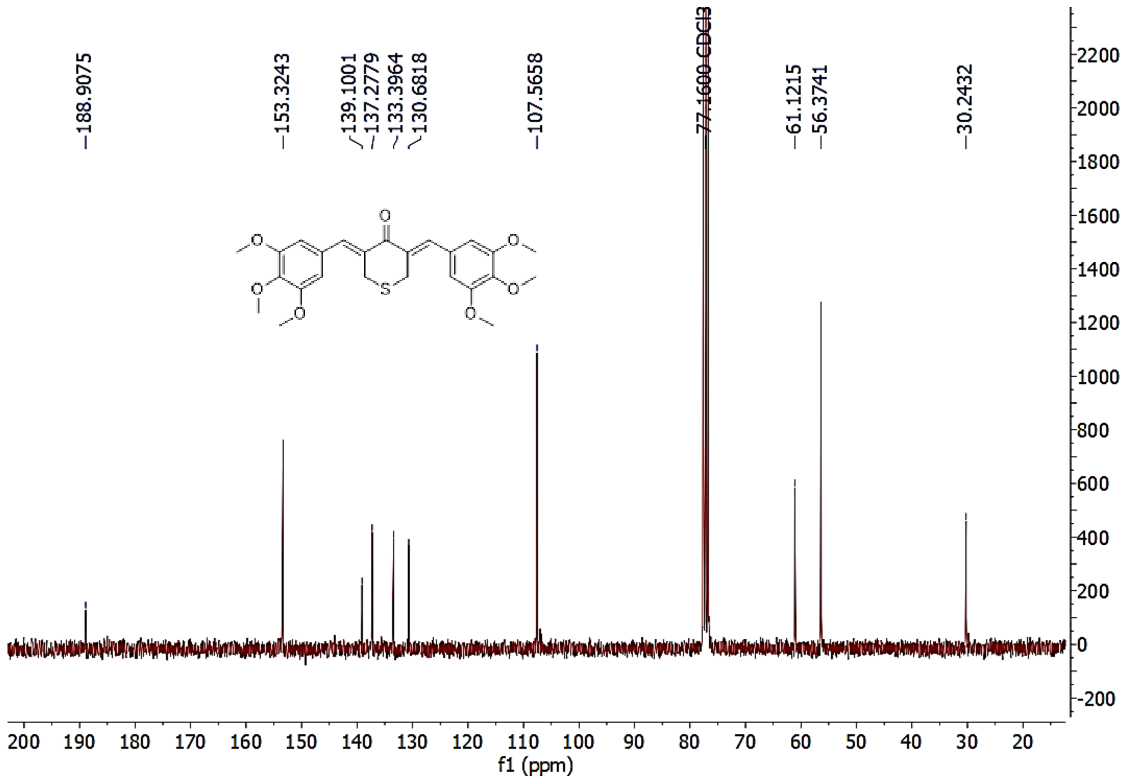

Figure S5. ^{1}H and ^{13}C NMR spectra of compound **9**.

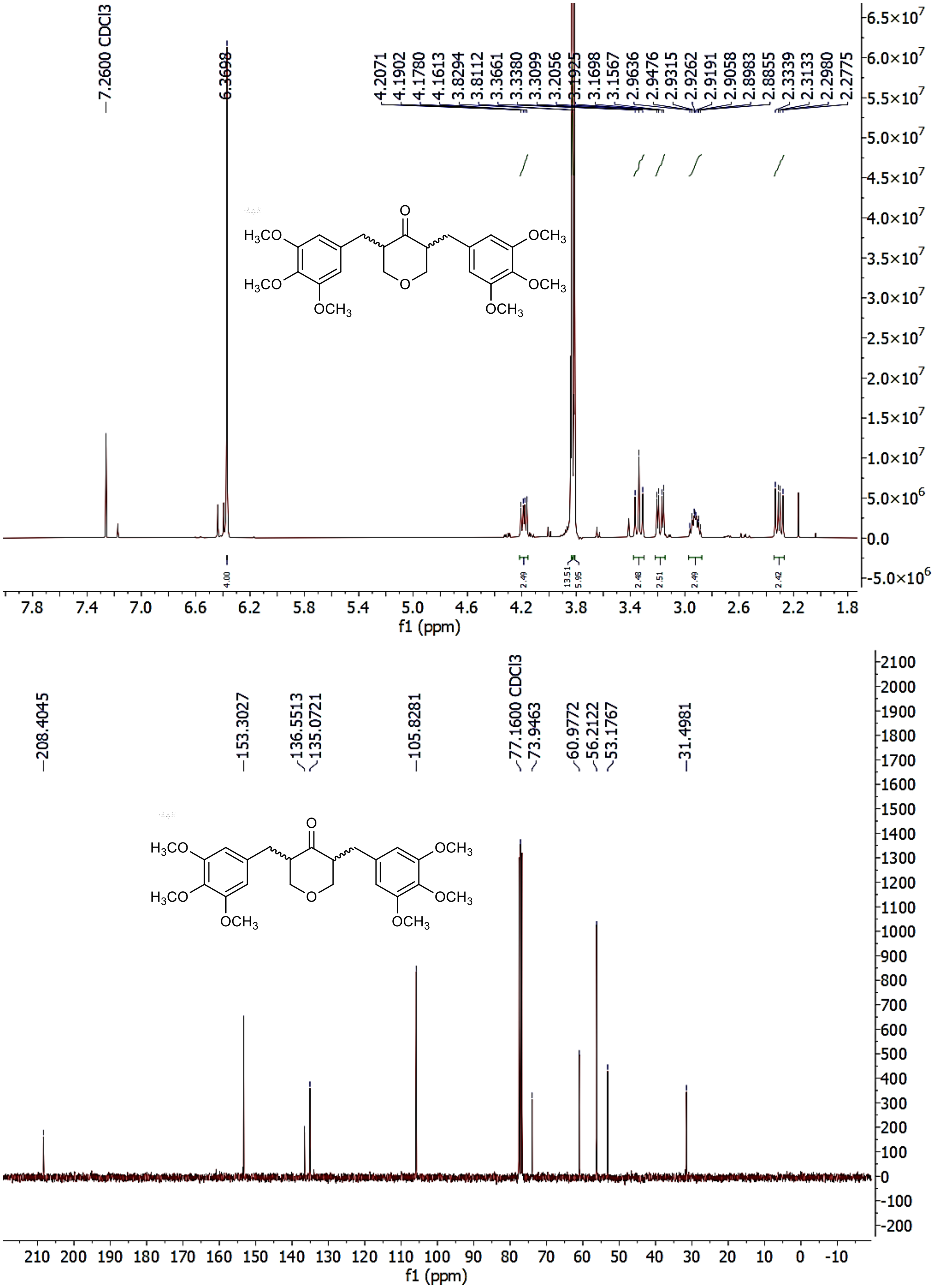

Figure S6. ^{1}H and ^{13}C NMR spectra of compound **10**.

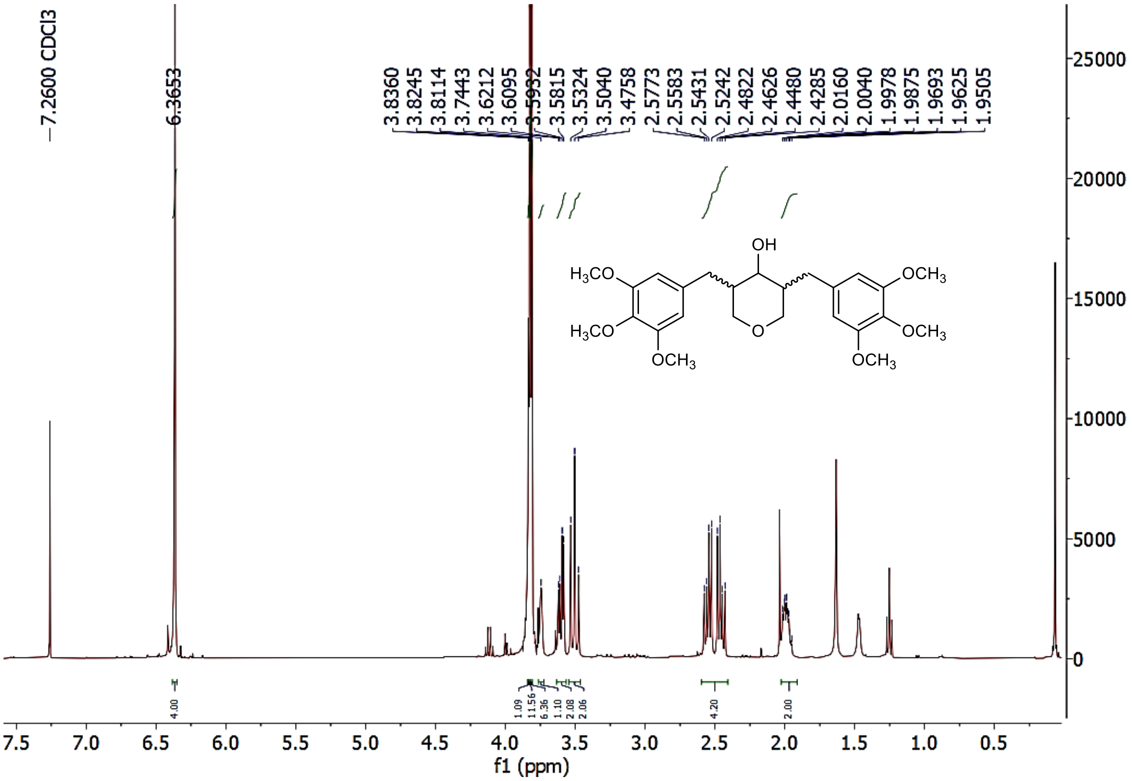

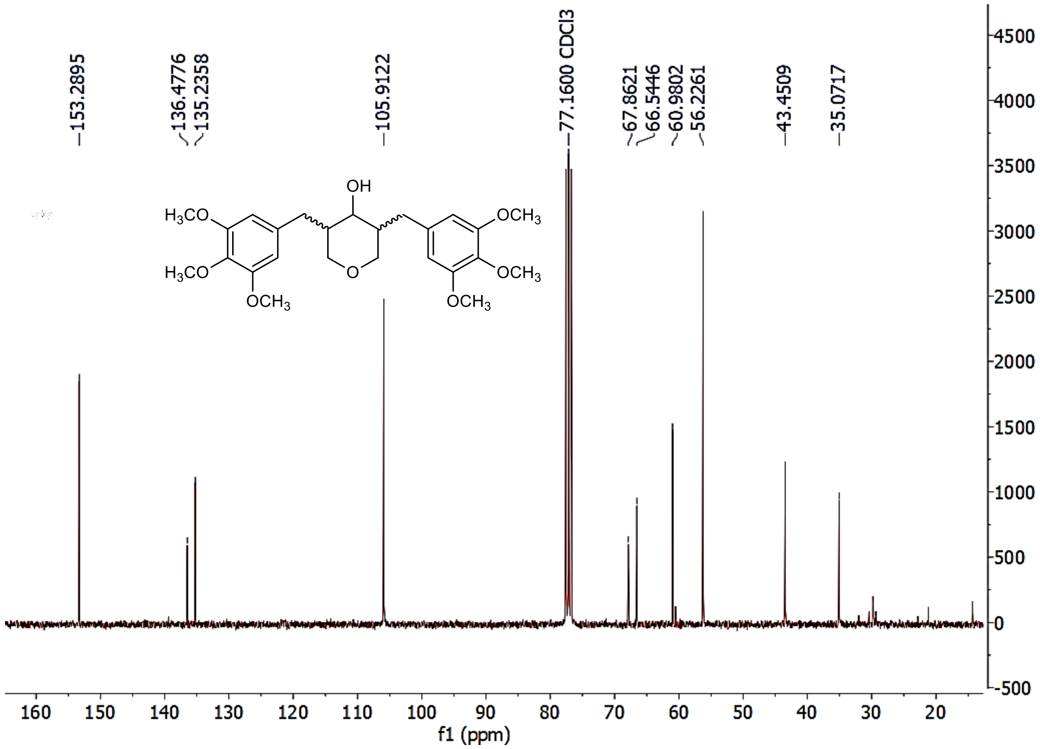

Figure S7. ^{1}H and ^{13}C NMR spectra of compound **11**.

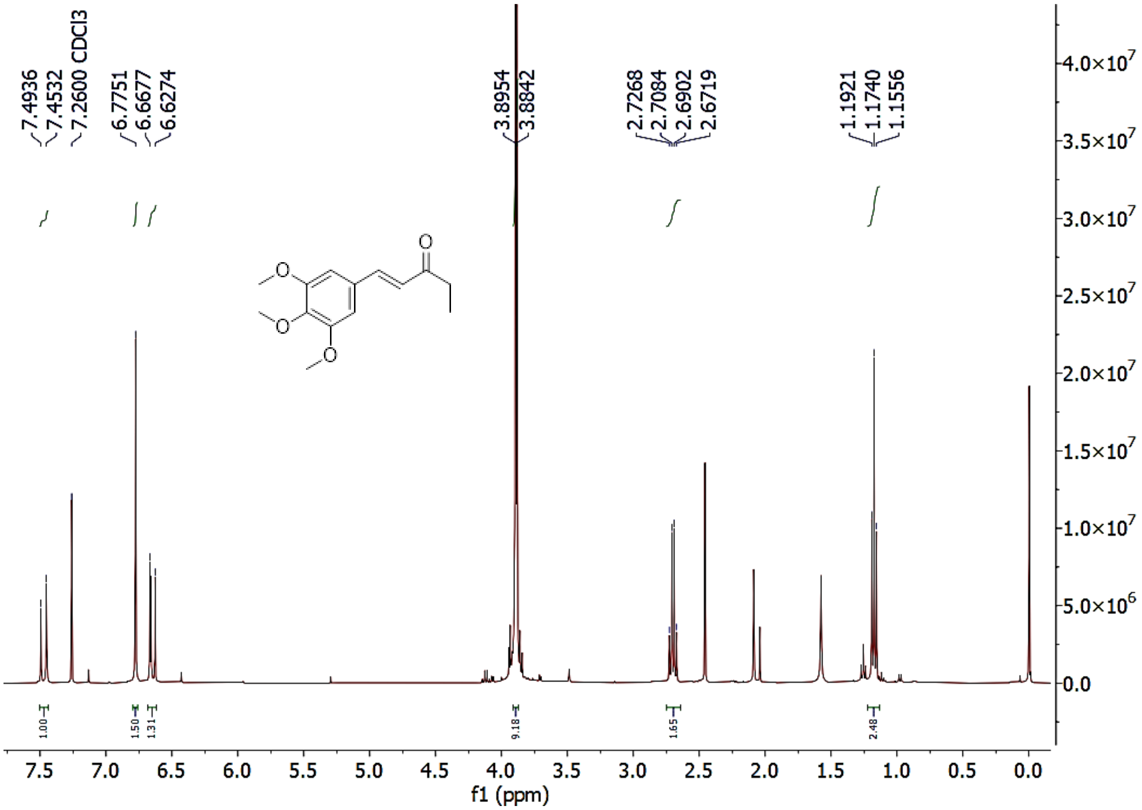

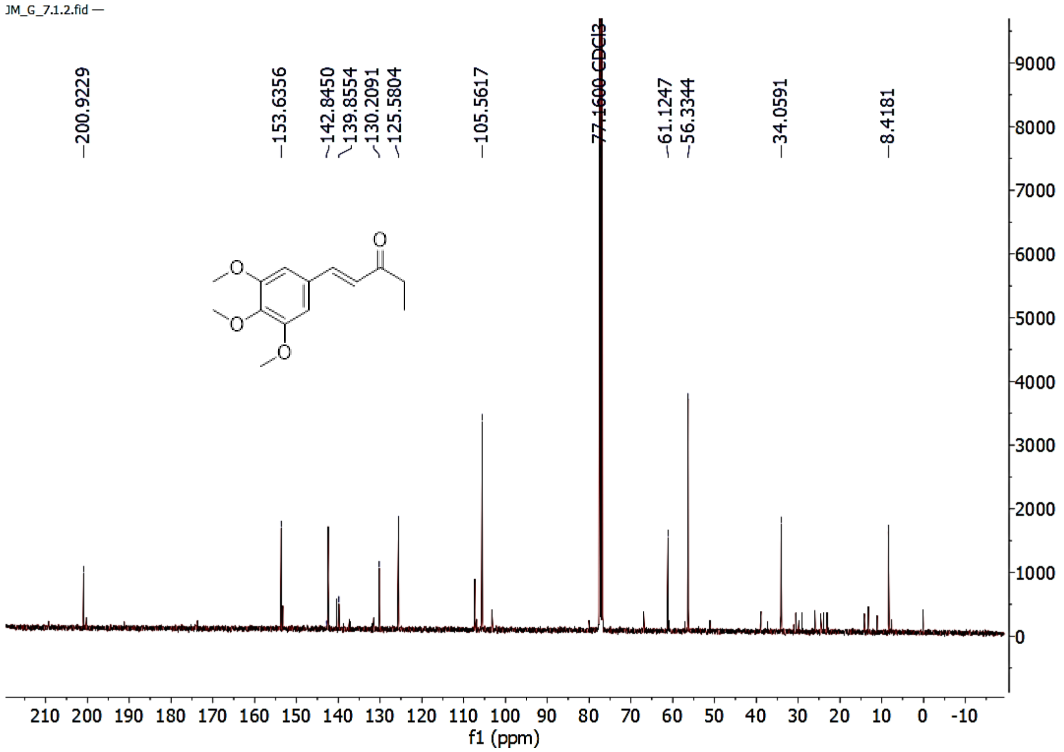

Figure S8. ^{1}H and ^{13}C NMR spectra of compound **12**.

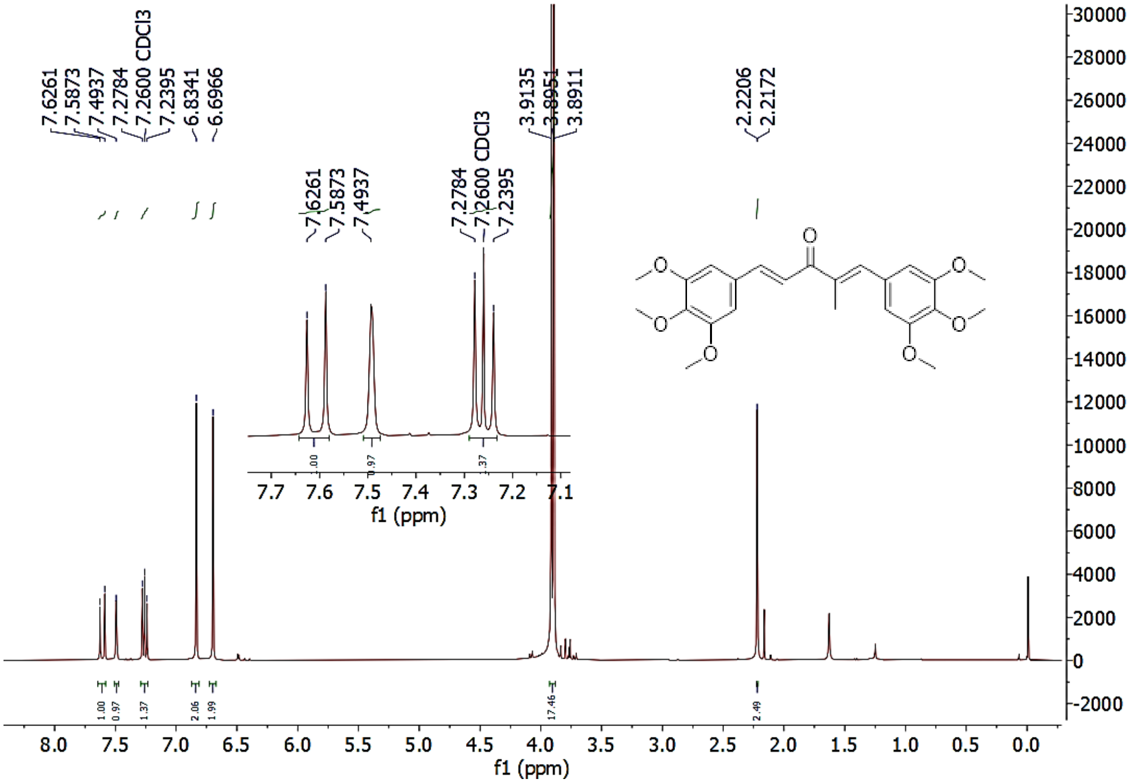

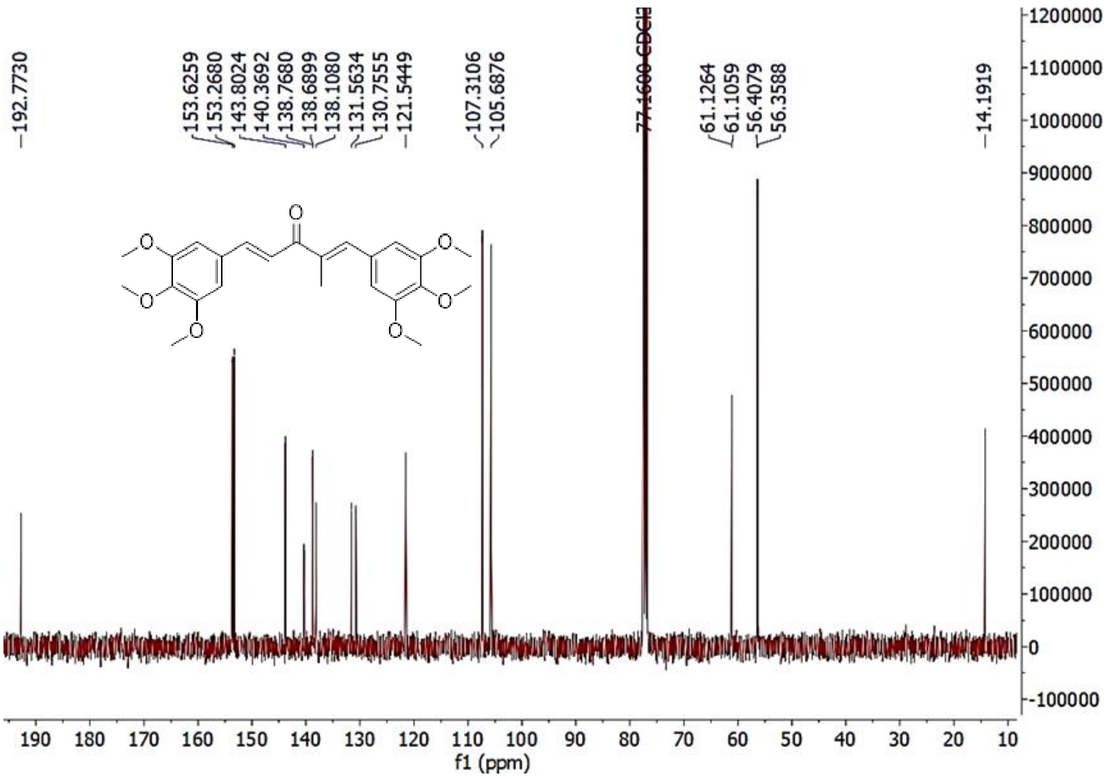

Figure S9. ^{1}H and ^{13}C NMR spectra of compound **13**.

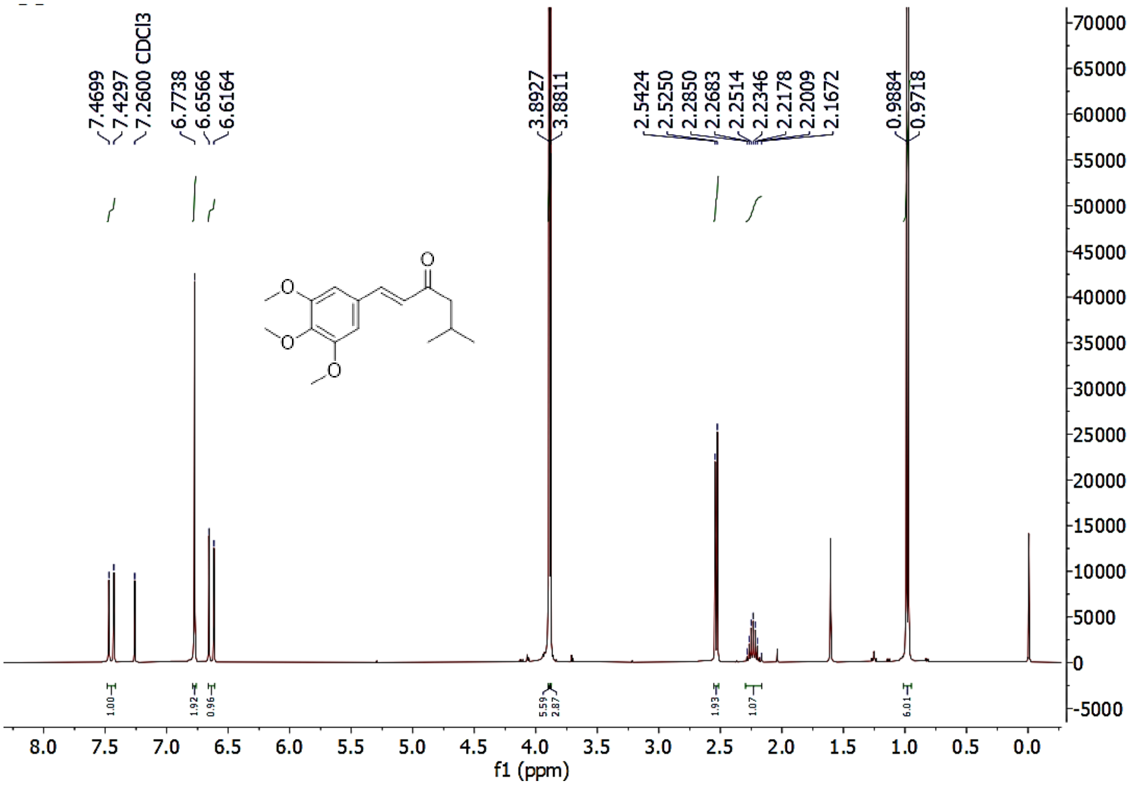

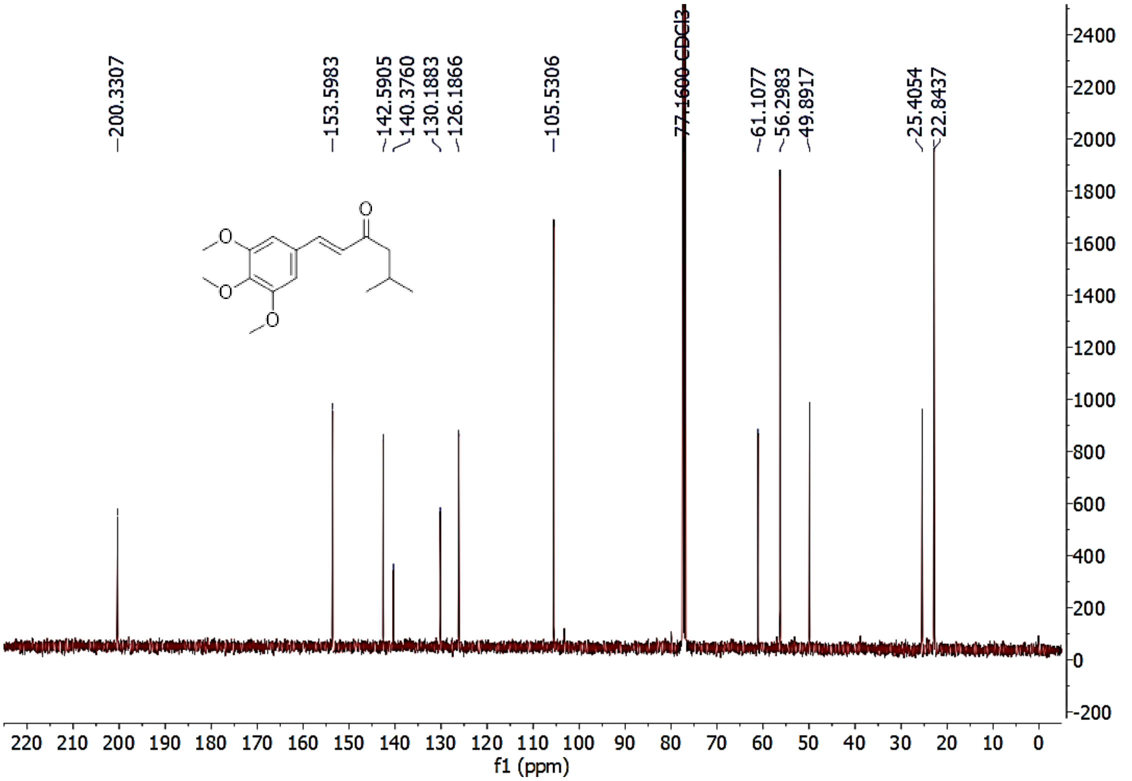

Figure S10. ^{1}H and ^{13}C NMR spectra of compound **14**.

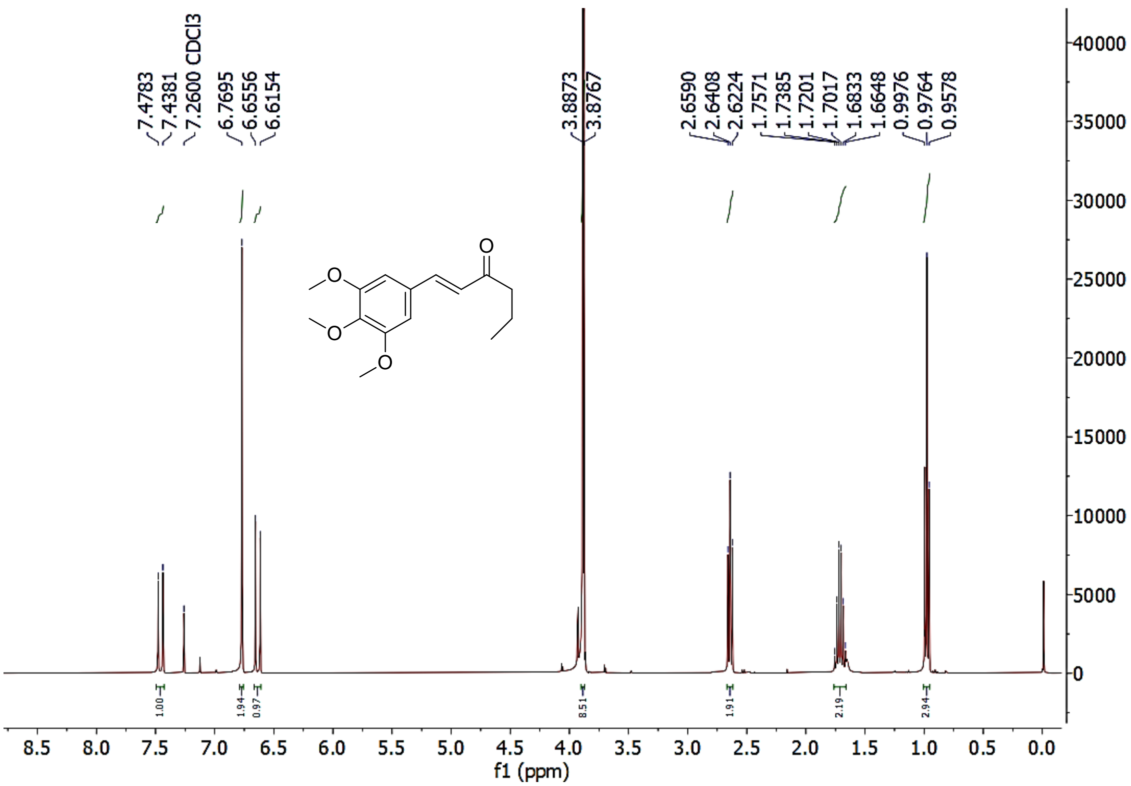

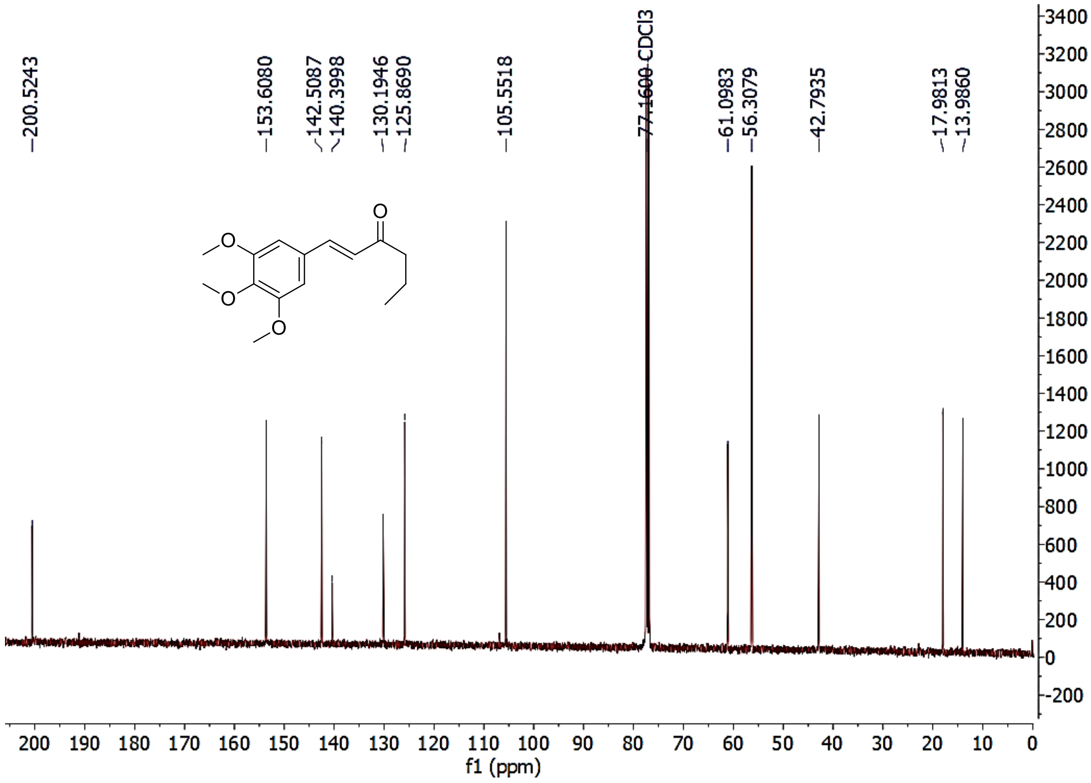

Figure S11. ^{1}H and ^{13}C NMR spectra of compound **15**.

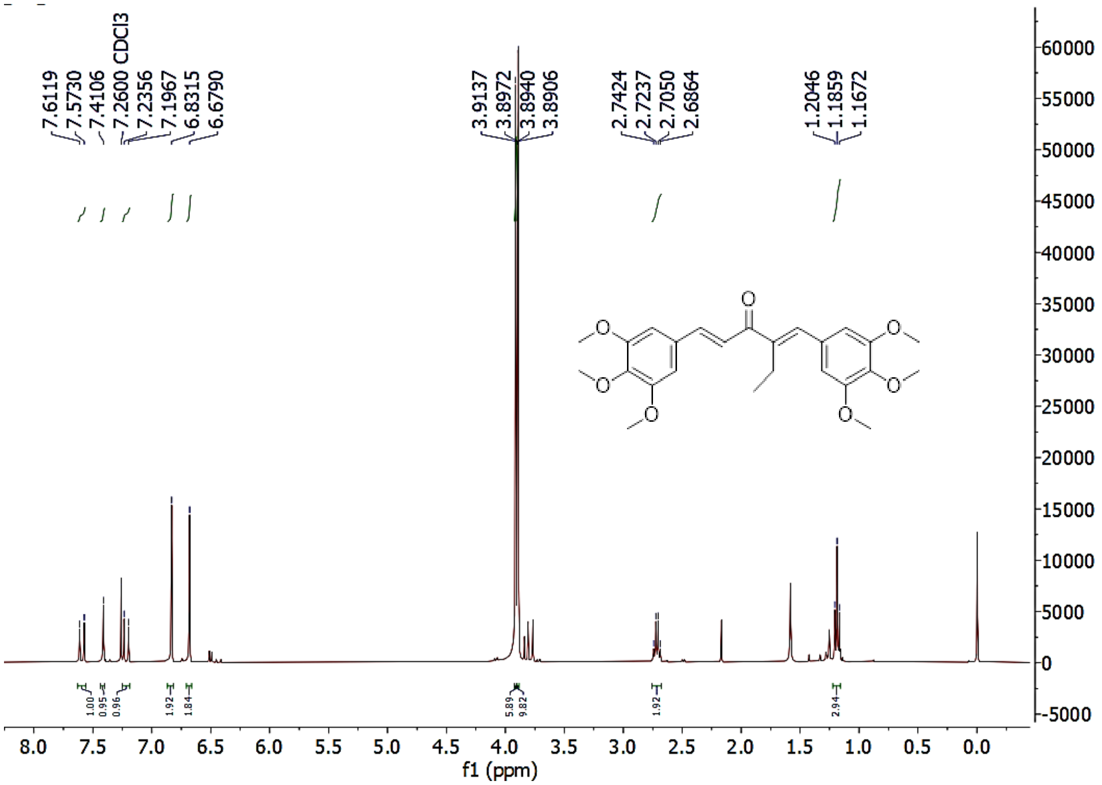

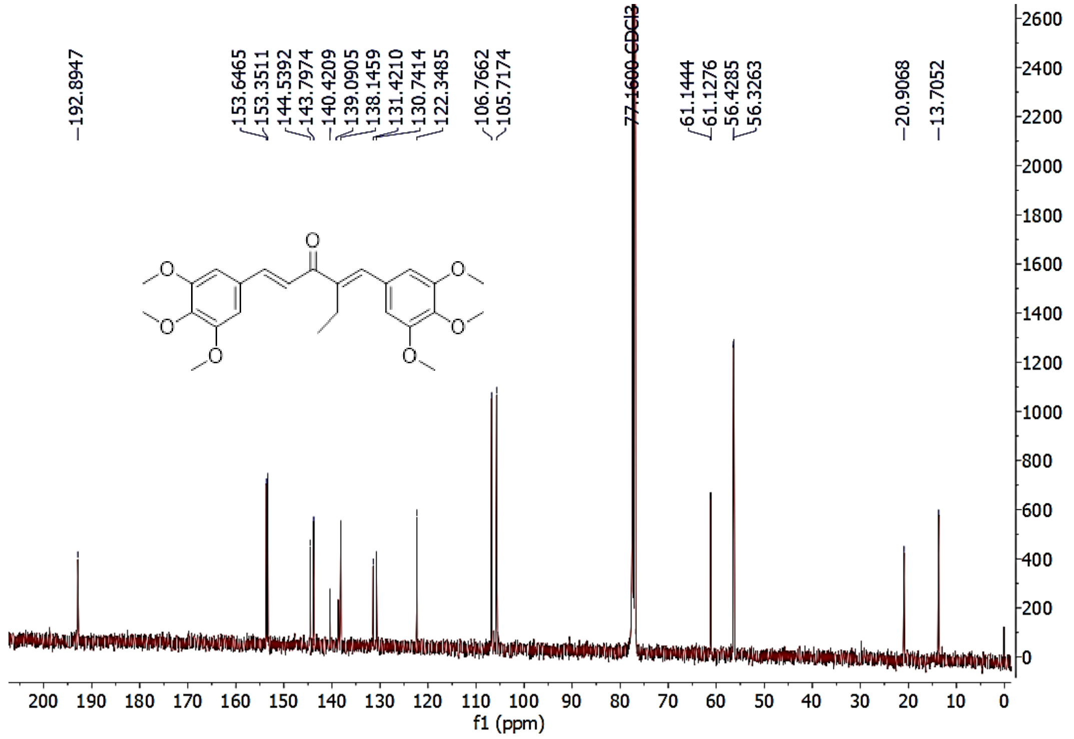

Figure S12. ^{1}H and ^{13}C NMR spectra of compound **16**.

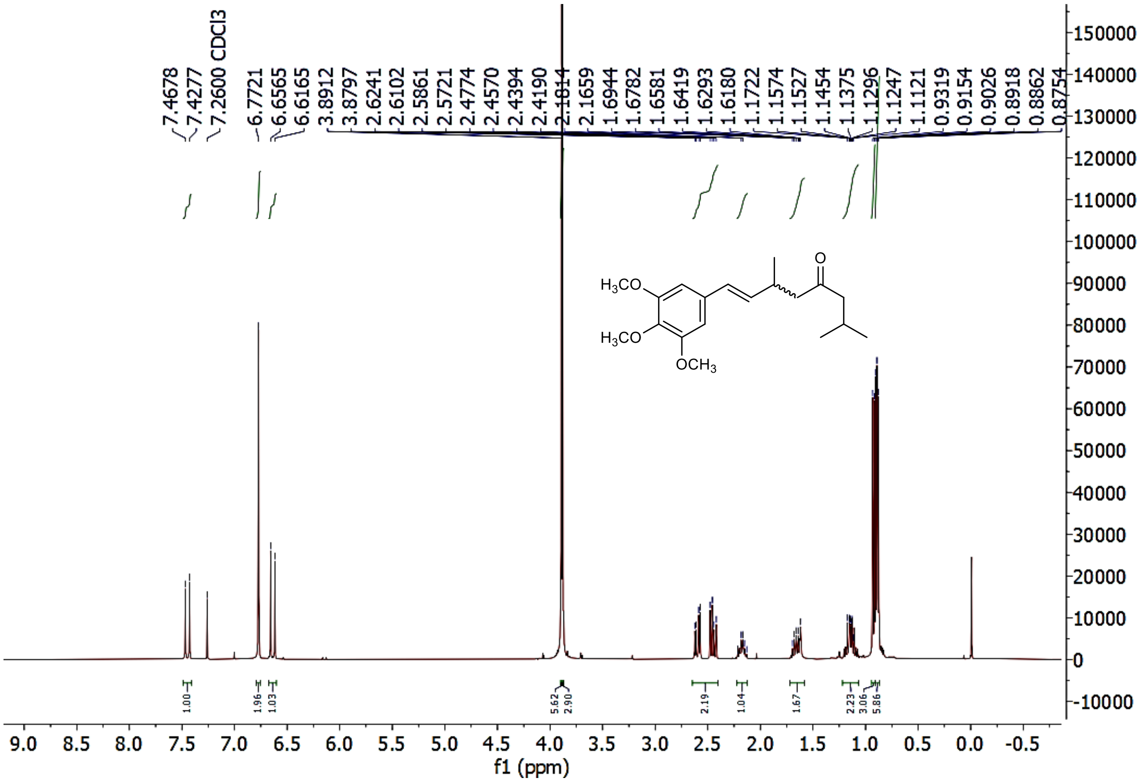

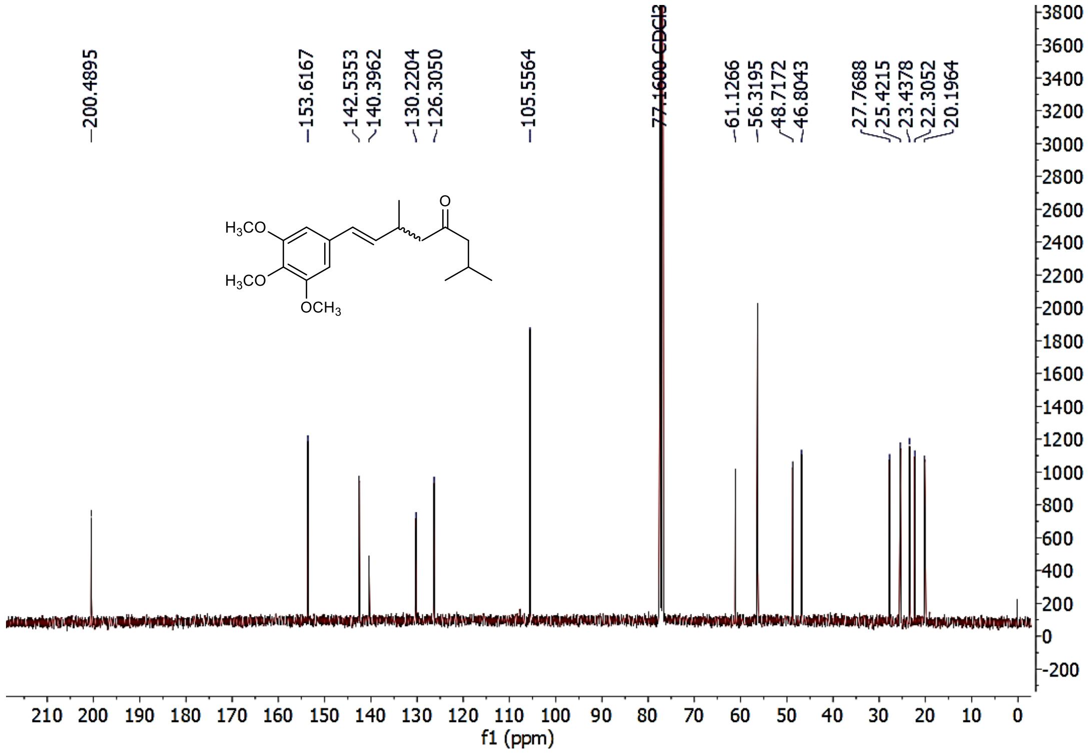

Figure S13. ^{1}H and ^{13}C NMR spectra of compound **17**.

HRMS spectra

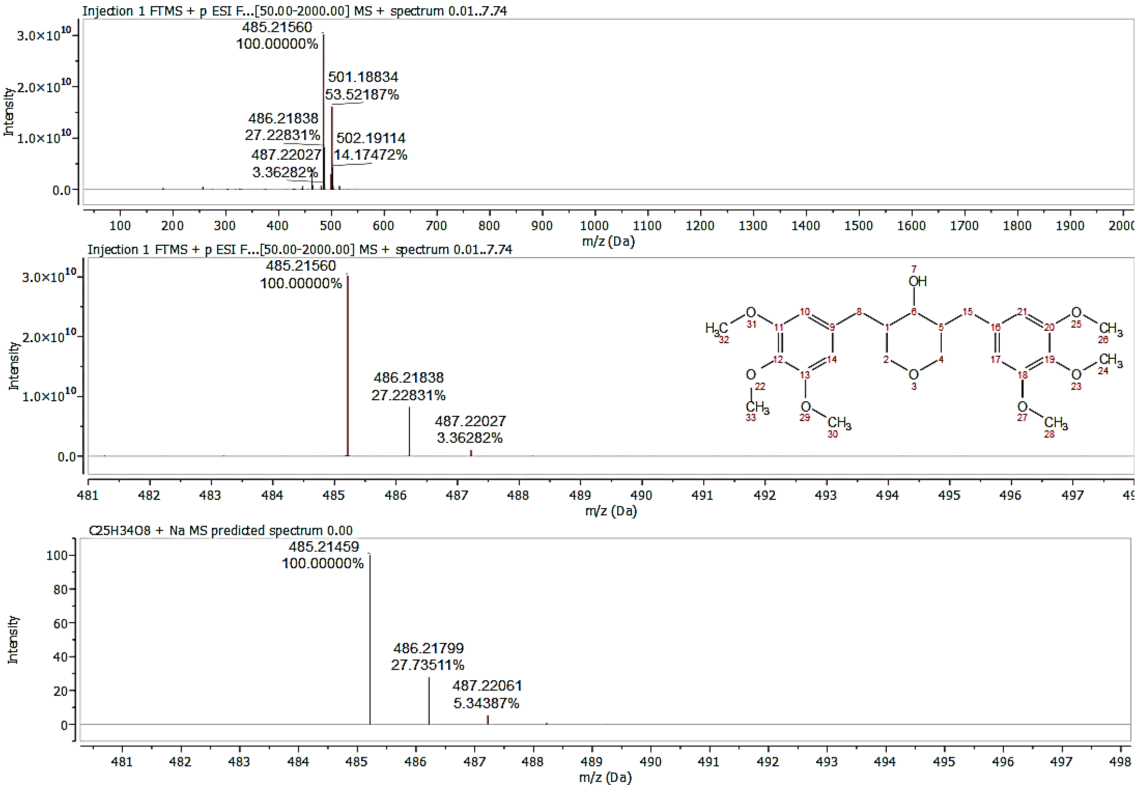

Meas. m/z	Ion formula	m/z	Err [ppm]	Err [mDa]
485.21560	$C_{25}H_{34}O_8$	485.21459	-2.08	-1.01

Figure S14. HRMS spectrum of compound **10**.

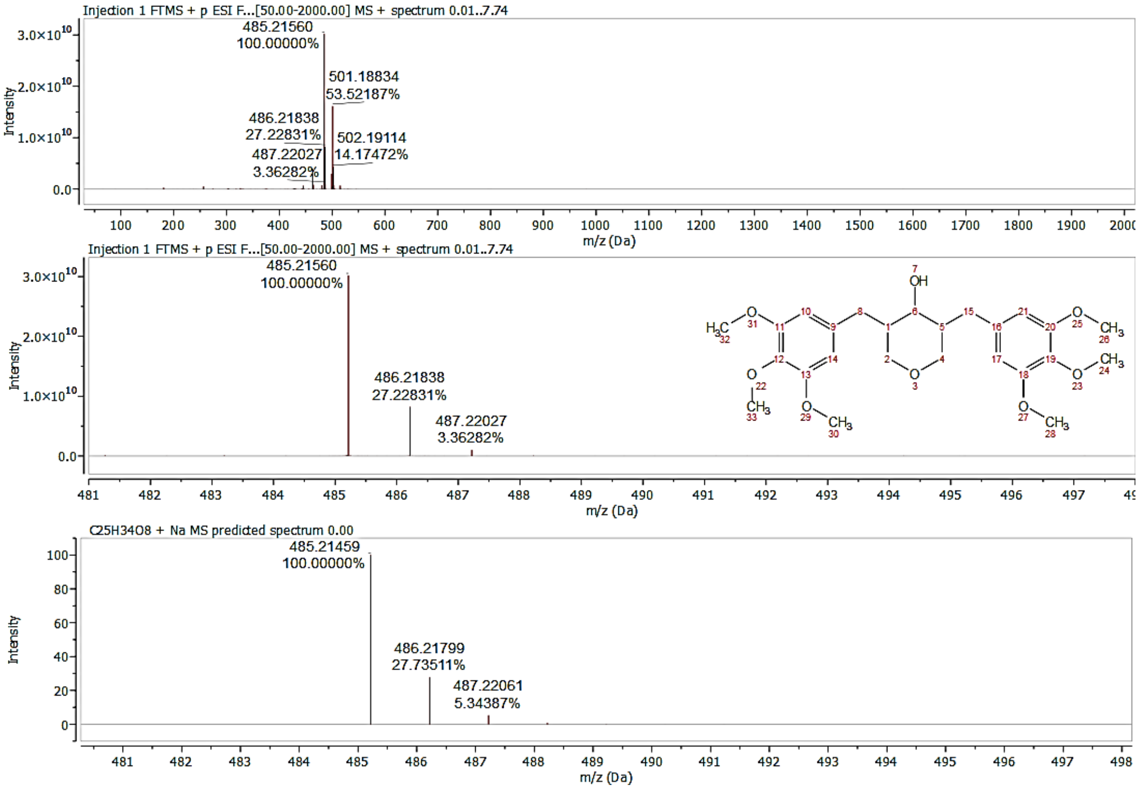

Meas. m/z	Ion formula	m/z	Err [ppm]	Err [mDa]
485.21560	$C_{25}H_{34}O_8$	485.21459	-2.08	-1.01

Figure S15. HRMS spectrum of compound **11**.

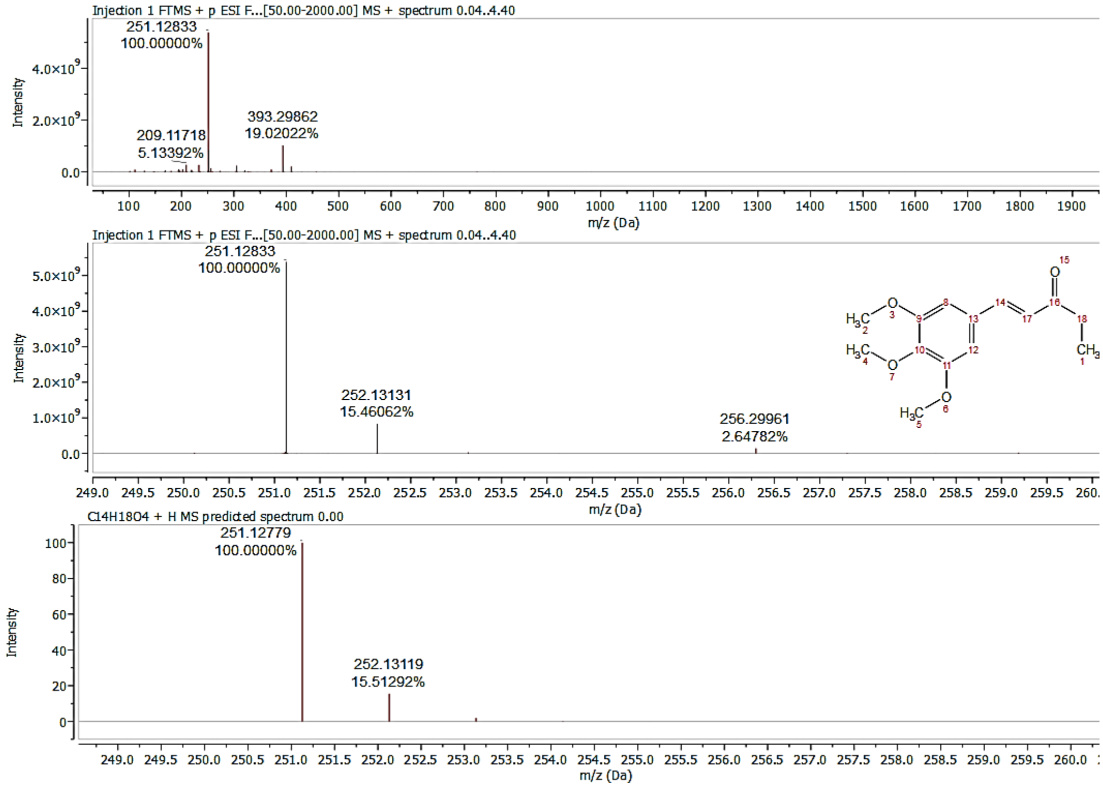

Meas. m/z	Ion formula	m/z	Err [ppm]	Err [mDa]
251.12833	$C_{14}H_{18}O_4$	251.12779	-2.16	-0.54

Figure S16. HRMS spectrum of compound **12**.

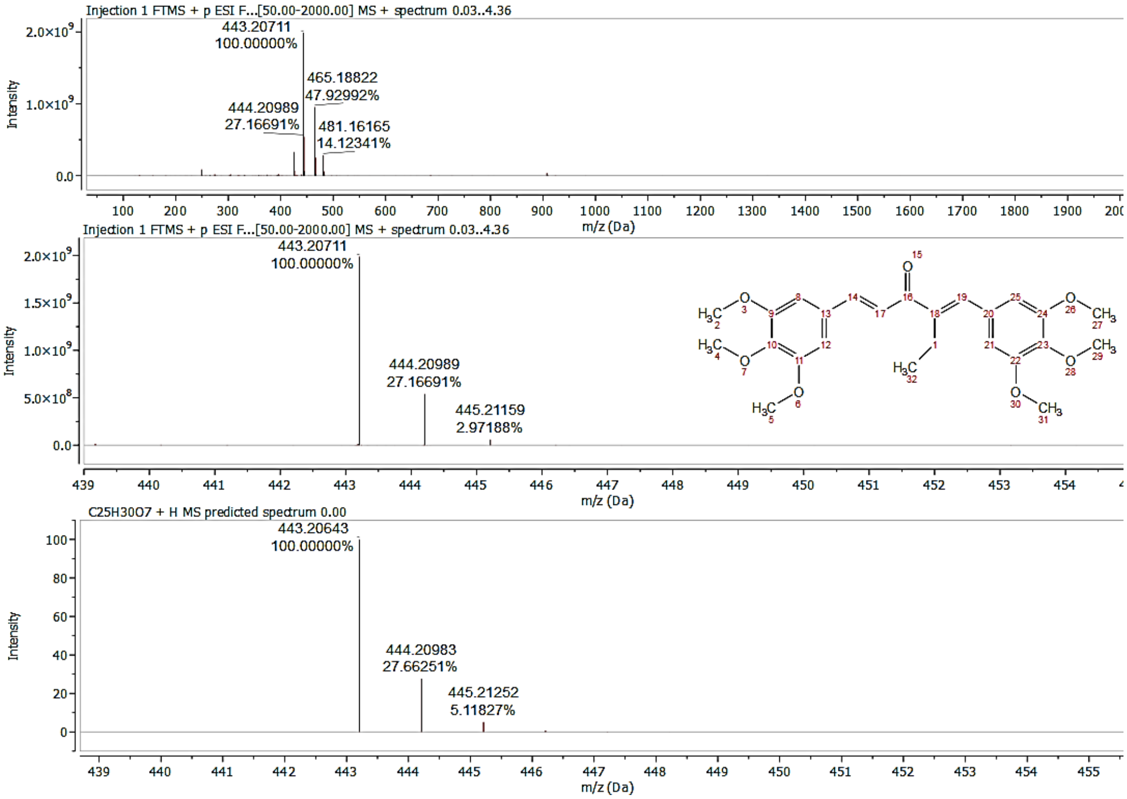

Meas. m/z	Ion formula	m/z	Err [ppm]	Err [mDa]
443.20711	$C_{25}H_{30}O_7$	443.20643	-1.53	-0.68

Figure S17. HRMS spectrum of compound **13**.

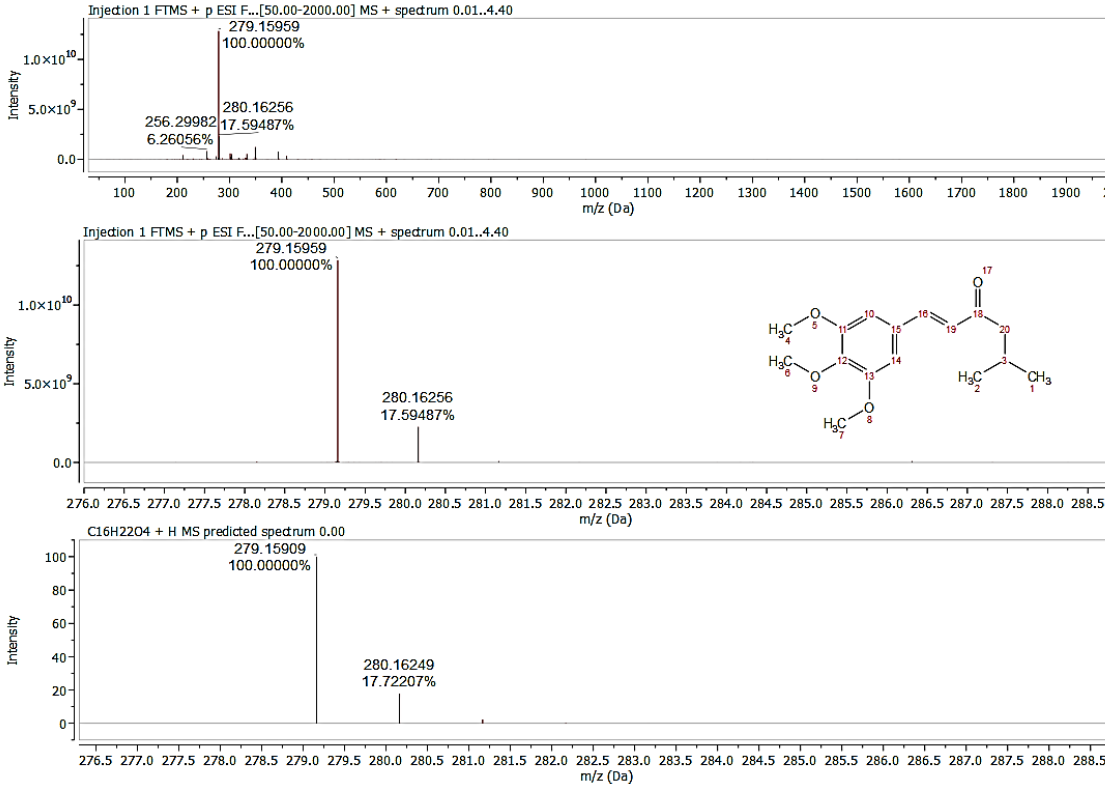

Meas. m/z	Ion formula	m/z	Err [ppm]	Err [mDa]
279.15959	$C_{16}H_{22}O_4$	279.15909	-1.80	-0.50

Figure S18. HRMS spectrum of compound **14**.

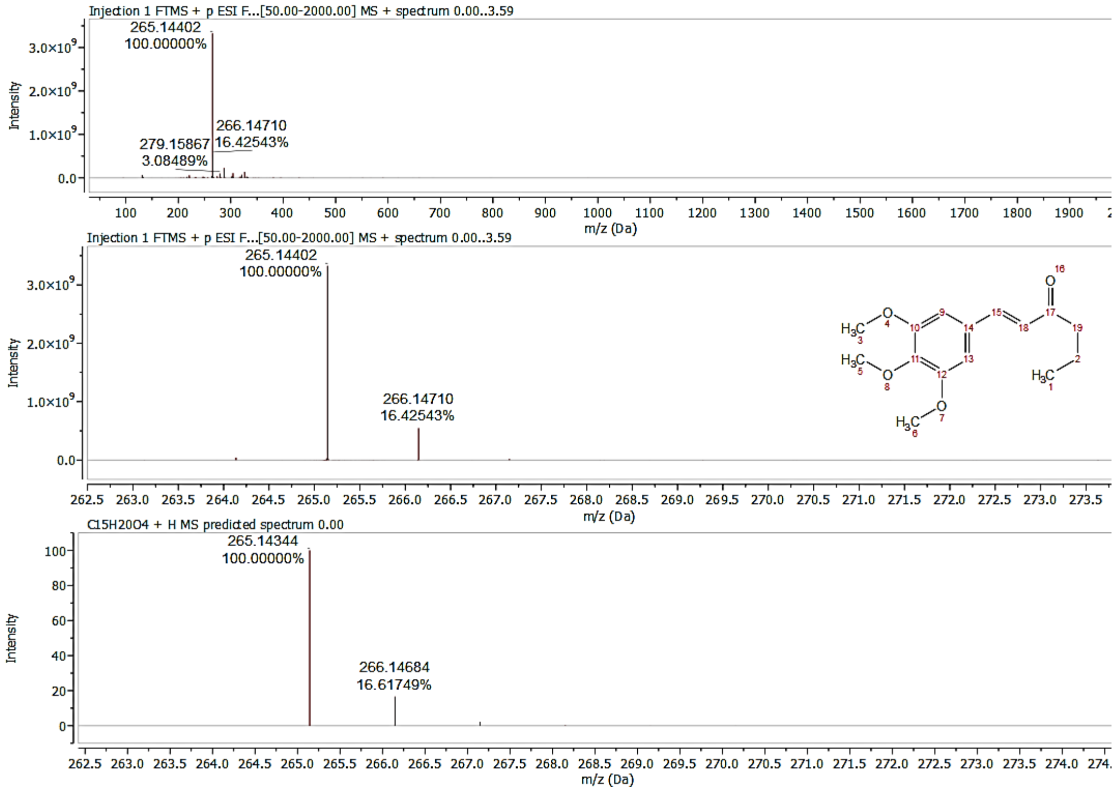

Meas. m/z	Ion formula	m/z	Err [ppm]	Err [mDa]
265.14402	$C_{15}H_{20}O_4$	265.14402	-2.20	-0.58

Figure S19. HRMS spectrum of compound **15**.

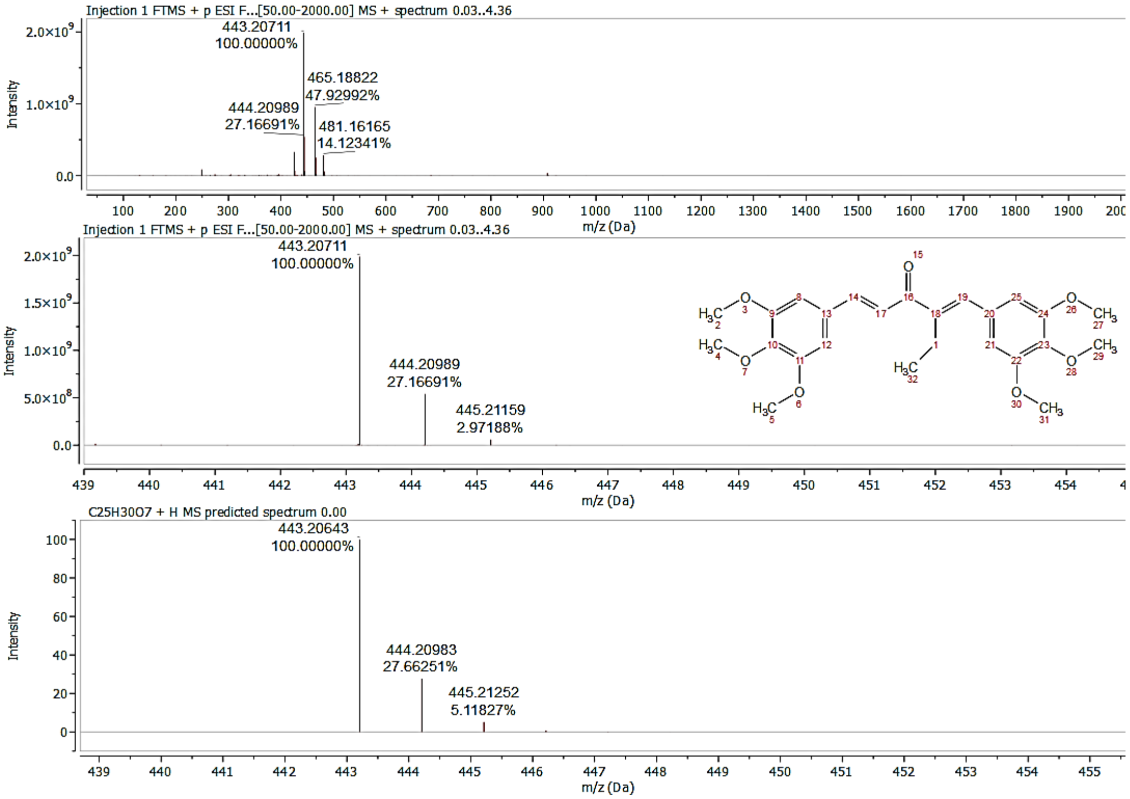

Meas. m/z	Ion formula	m/z	Err [ppm]	Err [mDa]
443.20711	$C_{25}H_{30}O_7$	443.20643	-1.53	-0.68

Figure S20. HRMS spectrum of compound **16**.

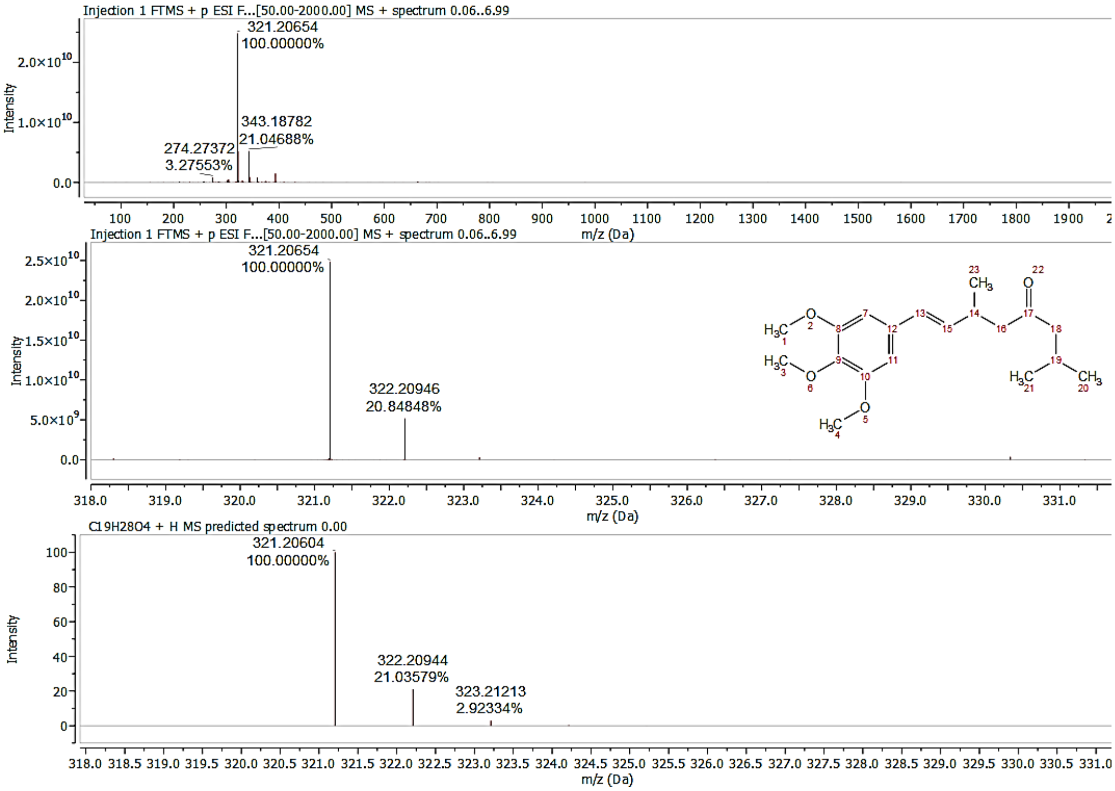

Meas. m/z	Ion formula	m/z	Err [ppm]	Err [mDa]
321.20654	$C_{19}H_{28}O_4$	321.20604	-1.57	-0.50

Figure S21. HRMS spectrum of compound **17**.

HPLC chromatograms

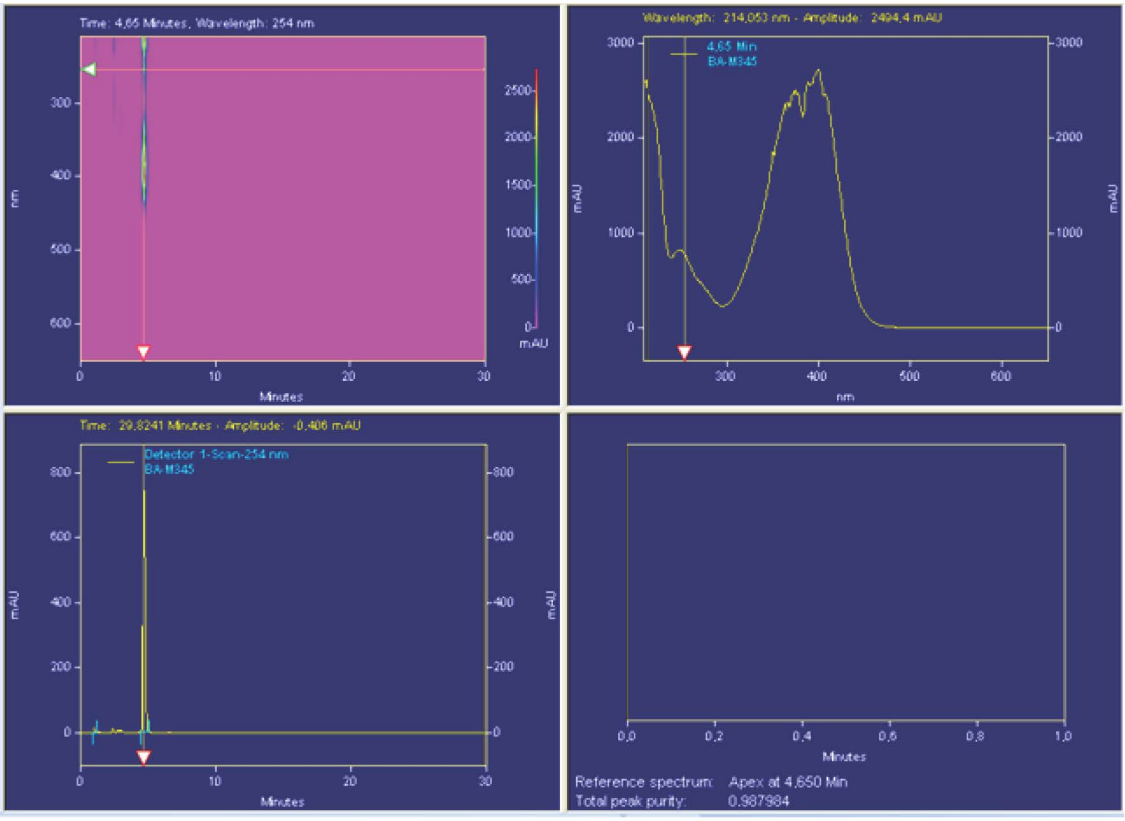

Retention Time	Area	Area %	Height	Height %
1.017	47698	0.45	17153	2.14
4.650	10511125	99.55	783993	97.86
Totals	1.017	47698	0.45	17153

Figure S22. HPLC chromatograms of compound **6**.

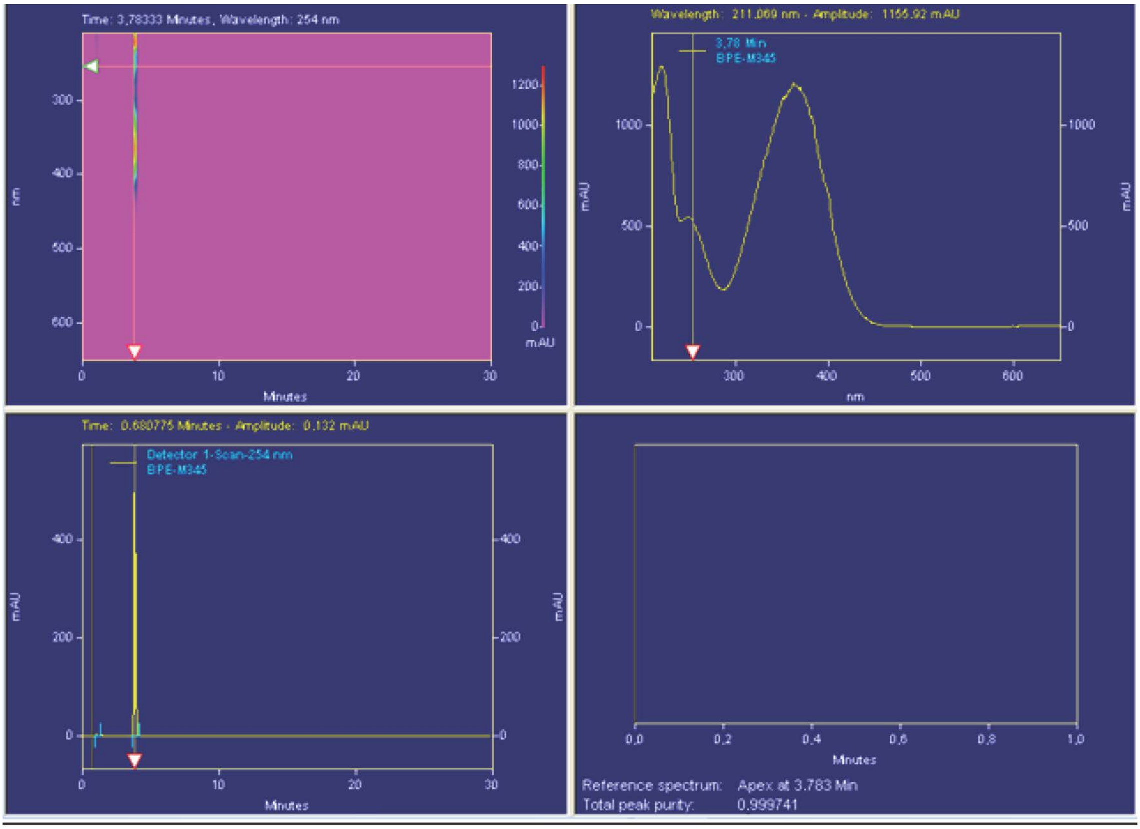

Retention Time	Area	Area %	Height	Height %
0.983	23362	0.41	6624	1.24
3.783	5716778	99.59	526936	98.76
Totals	5740140	100.00	533560	100.00

Figure S23. HPLC chromatograms of compound **7**.

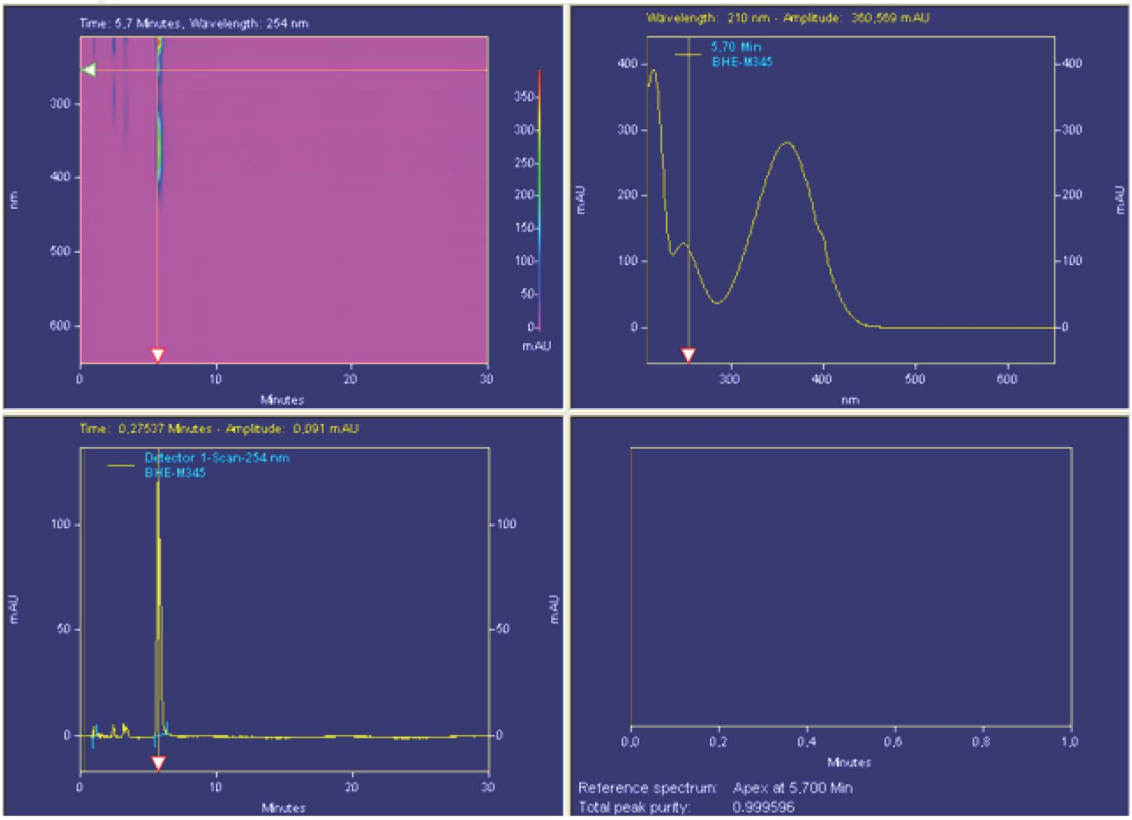

Retention Time	Area	Area %	Height	Height %
0.967	21613	1.03	5243	4.17
5.700	2069200	98.97	120581	95.83
Totals	2090813	100.00	125824	100.00

Figure S24. HPLC chromatograms of compound **8**.

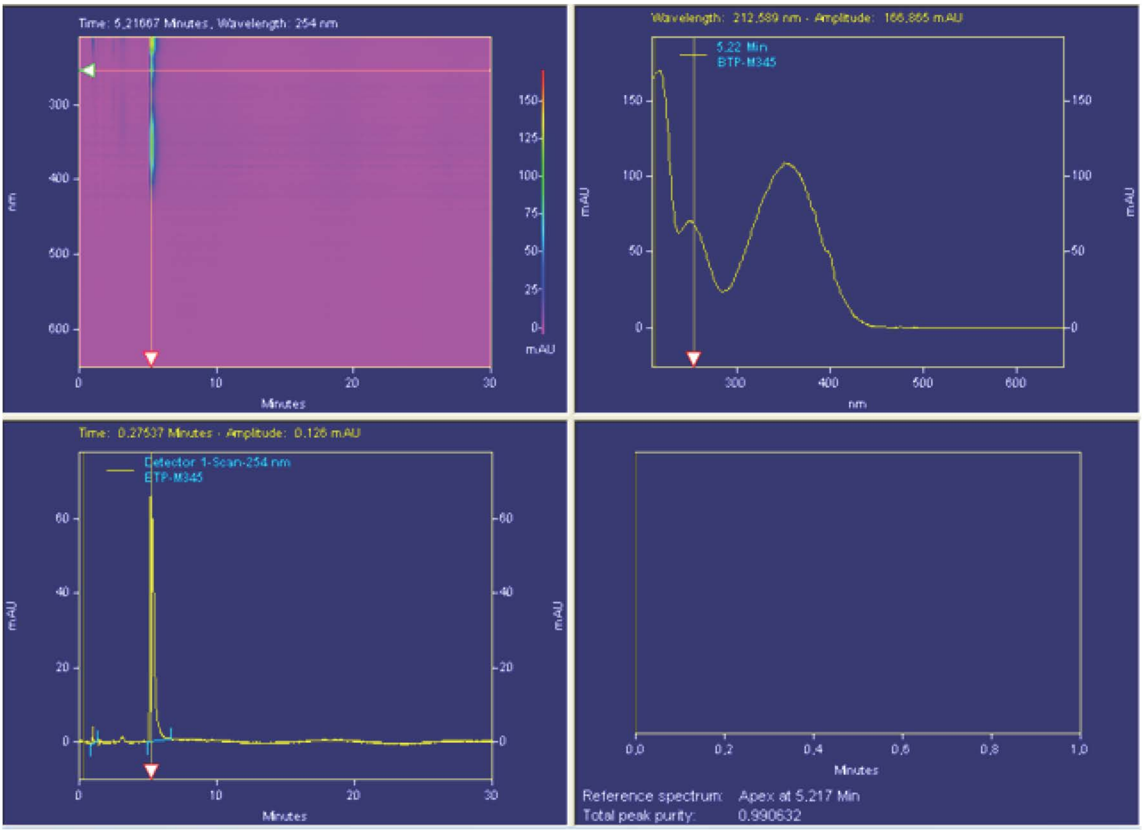

Retention Time	Area	Area %	Height	Height %
0.967	20869	1.51	4685	6.35
5.217	1362960	98.49	69143	93.65
Totals	1383829	100.00	73828	100.00

Figure S25. HPLC chromatograms of compound **9**.

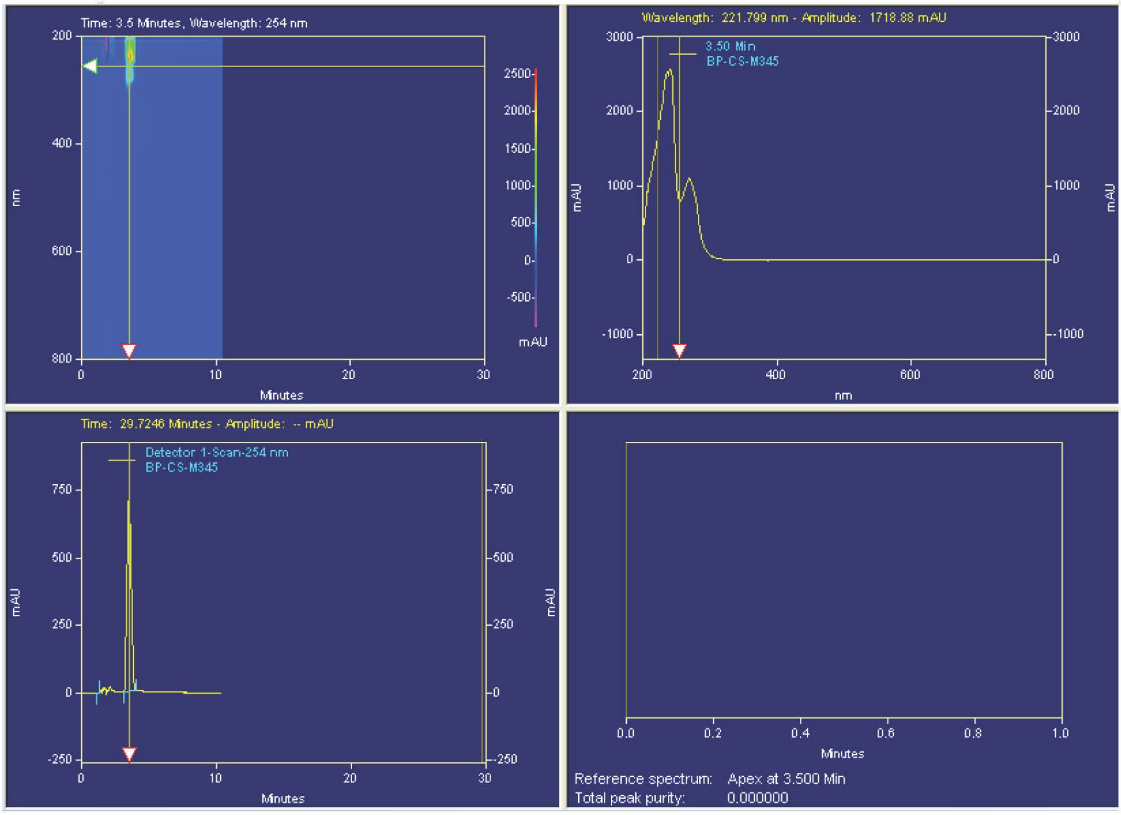

Retention Time	Area	Area %	Height	Height %
1.217	5302	0.03	905	0.11
3.500	16369382	99.97	803807	99.89
Totals	16374684	100.00	804712	100.00

Figure S26. HPLC chromatograms of compound **10**.

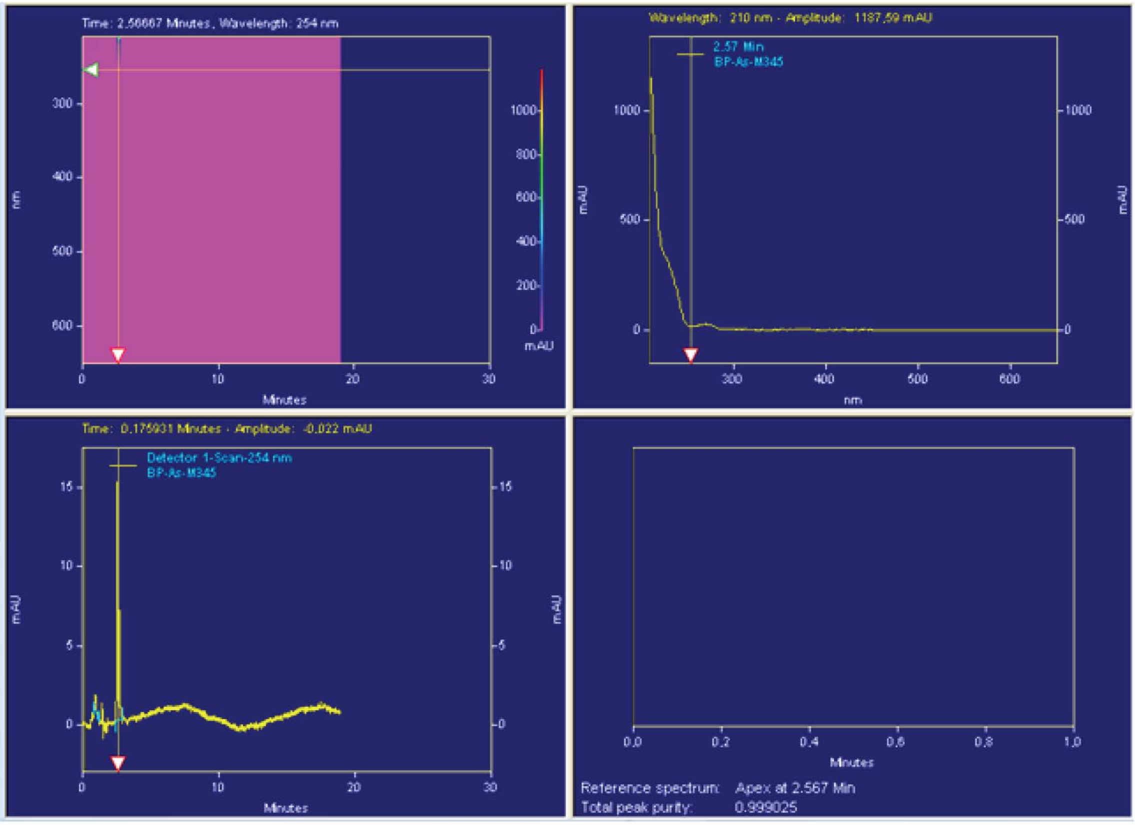

Retention Time	Area	Area %	Height	Height %
0.933	6986	4.56	888	5.50
2.567	146350	95.44	15248	94.50
Totals	153336	100.00	16136	100.00

Figure S27. HPLC chromatograms of compound **11**.

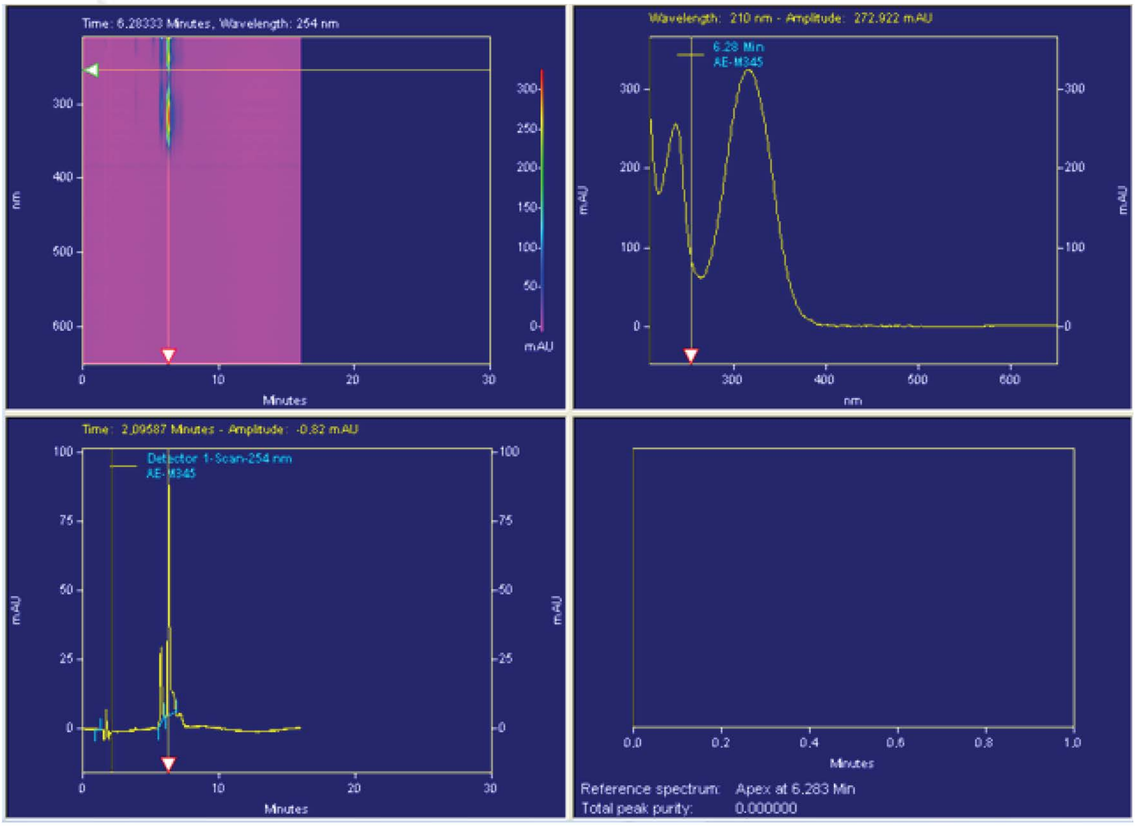

Retention Time	Area	Area %	Height	Height %
1.033	4002	0.32	323	0.29
6.283	1237422	99.68	111914	99.71
Totals	1241424	100.00	112237	100.00

Figure S28. HPLC chromatograms of compound **12**.

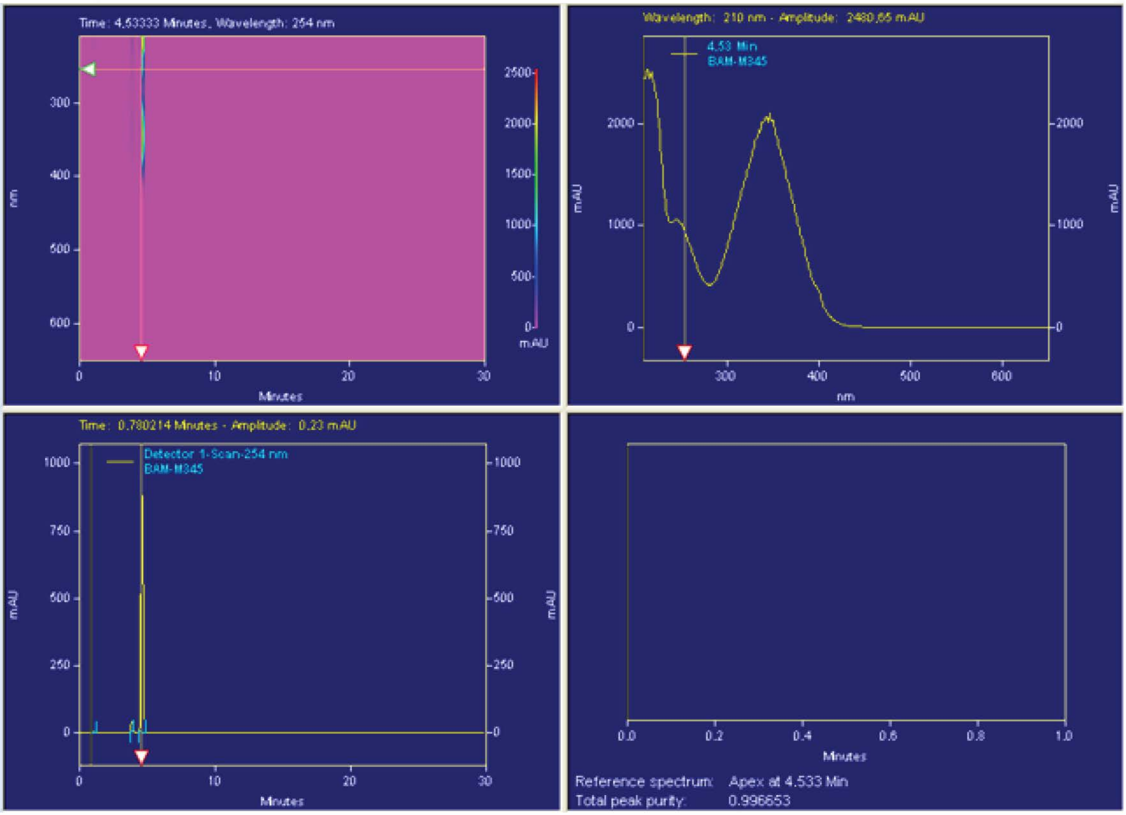

Retention Time	Area	Area %	Height	Height %
0.950	18713	0.15	4596	0.47
3.800	326562	2.64	34631	3.51
4.533	12025722	97.21	947850	96.03
Totals	12370997	100.00	987077	100.00

Figure S29. HPLC chromatograms of compound **13**.

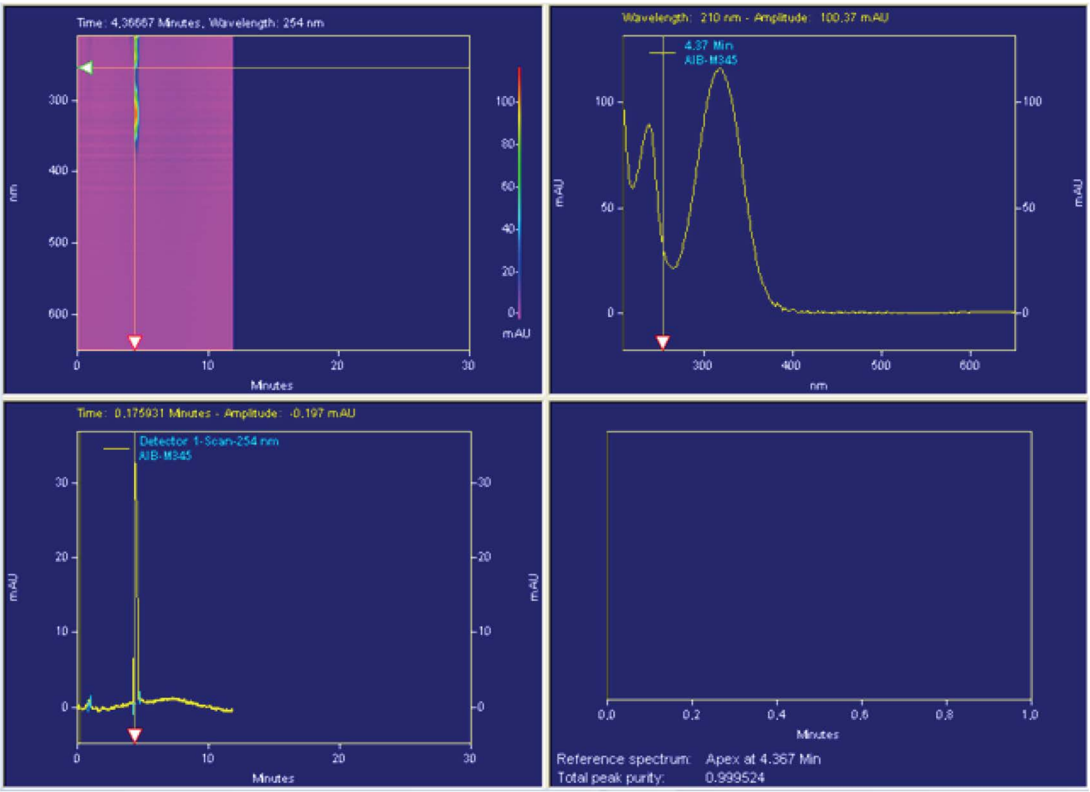

Retention Time	Area	Area %	Height	Height %
0.900	2301	0.47	403	1.23
4.367	490186	99.53	32268	98.77
Totals	492487	100.00	32671	100.00

Figure S30. HPLC chromatograms of compound **14**.

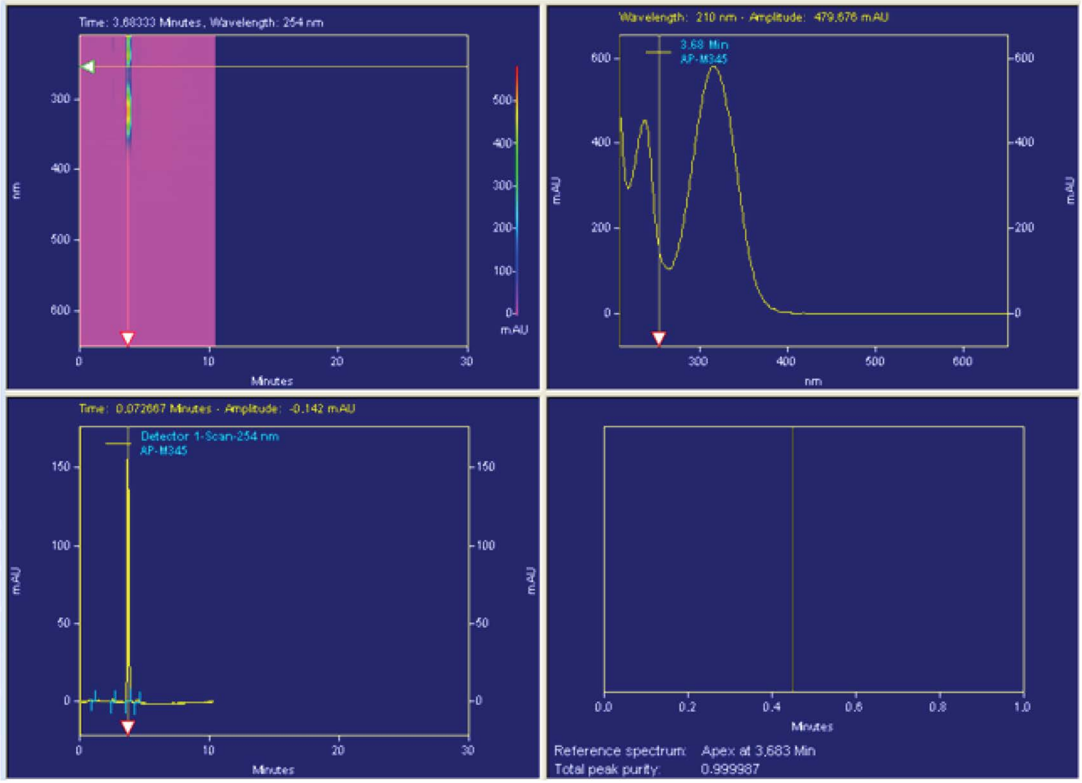

Retention Time	Area	Area %	Height	Height %
0.883	3137	0.15	375	0.23
2.517	14648	0.72	1683	1.05
3.683	1997569	97.84	156372	97.55
4.400	26335	1.29	1870	1.17
Totals	2041689	100.00	160300	100.00

Figure S31. HPLC chromatograms of compound **15**.

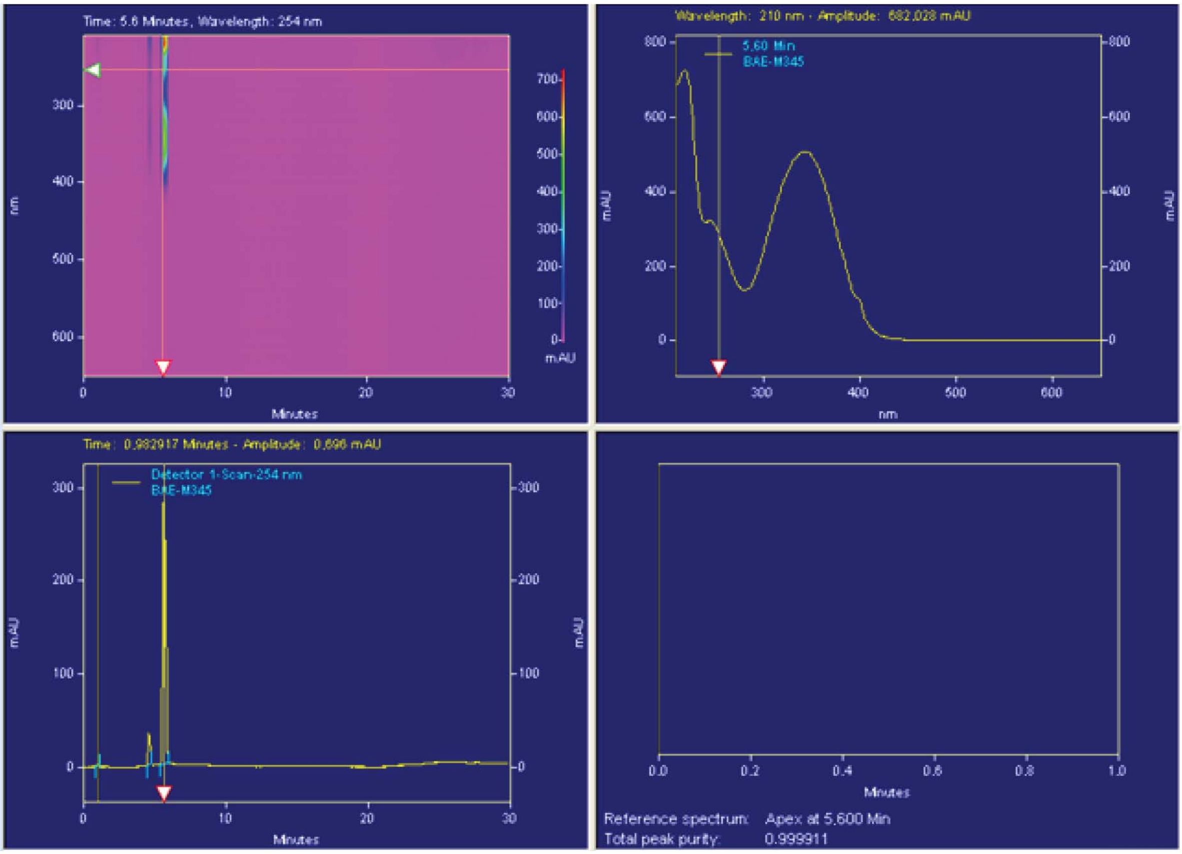

Retention Time	Area	Area %	Height	Height %
0.917	9773	0.20	1827	0.57
4.550	206343	4.22	16991	5.28
5.600	4670834	95.58	302936	94.09
Totals	4886950	100.00	321754	100.00

Figure S32. HPLC chromatograms of compound **16**.

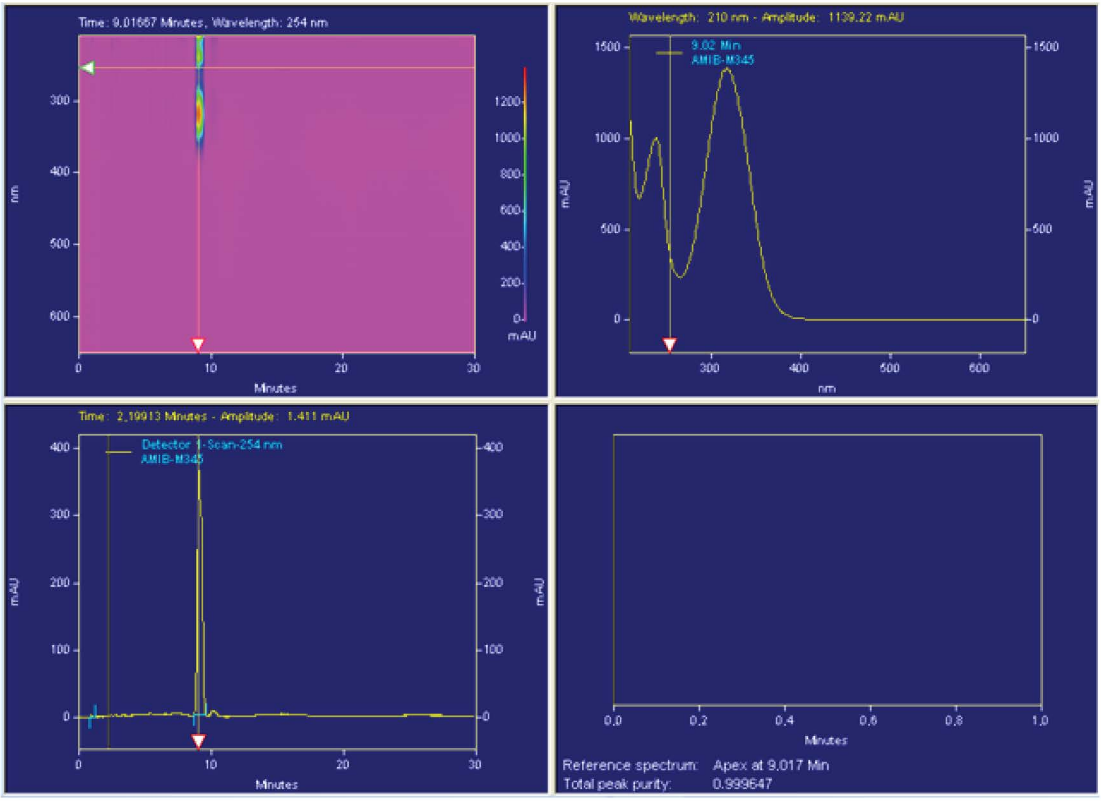

Retention Time	Area	Area %	Height	Height %
0.933	15505	0.17	3237	0.87
9.017	9133883	99.83	369124	99.13
Totals	9149388	100.00	372361	100.00

Figure S33. HPLC chromatograms of compound **17**.
